# Associations Between Maternal Distress During Early Life Periods and Offspring Respiratory Infections and Allergic Outcomes

**DOI:** 10.3389/fped.2022.749323

**Published:** 2022-03-30

**Authors:** Hui Xing Lau, Michelle Zhi Ling Kee, Qai Ven Yap, Elizabeth Huiwen Tham, Yiong Huak Chan, Anne Eng Neo Goh, Oon Hoe Teoh, Johan Gunnar Eriksson, Keith M. Godfrey, Peter D. Gluckman, Yap Seng Chong, Jerry Kok Yen Chan, Hugo Van Bever, Bee Wah Lee, Lynette Pei-chi Shek, Michael J. Meaney, Evelyn Xiu Ling Loo

**Affiliations:** ^1^Singapore Institute for Clinical Sciences (SICS), Agency for Science, Technology and Research (A^*^STAR), Singapore, Singapore; ^2^Department of Biostatistics, Yong Loo Lin School of Medicine, National University of Singapore, Singapore, Singapore; ^3^Department of Paediatrics, Yong Loo Lin School of Medicine, National University of Singapore, Singapore, Singapore; ^4^Khoo Teck Puat-National University Children's Medical Institute, National University Hospital, National University Health System, Singapore, Singapore; ^5^Human Potential Translational Research Programme, Yong Loo Lin School of Medicine, National University of Singapore, Singapore, Singapore; ^6^Department of Paediatrics, KK Women's and Children's Hospital, Singapore, Singapore; ^7^Department of Obstetrics & Gynaecology, Yong Loo Lin School of Medicine, National University of Singapore and National University Health System, Singapore, Singapore; ^8^Folkhälsan Research Center, Helsinki, Finland; ^9^Department of General Practice and Primary Health Care, University of Helsinki, Helsinki, Finland; ^10^MRC Lifecourse Epidemiology Unit and NIHR Southampton Biomedical Research Centre, University of Southampton and University Hospital Southampton NHS Foundation Trust, Southampton, United Kingdom; ^11^Liggins Institute, University of Auckland, Auckland, New Zealand; ^12^Department of Reproductive Medicine, KK Women's and Children's Hospital, Singapore, Singapore; ^13^Academic Program in Obstetrics and Gynaecology, Duke-NUS Medical School, Singapore, Singapore; ^14^Department of Psychiatry, Douglas Mental Health University Institute, McGill University, Montreal, QC, Canada; ^15^Sackler Program for Epigenetics and Psychobiology, McGill University, Montreal, QC, Canada; ^16^Ludmer Centre for Neuroinformatics and Mental Health, McGill University, Montréal, QC, Canada

**Keywords:** maternal distress, wheeze, rhinitis, eczema, allergic sensitization, preconception, pregnancy, postnatal

## Abstract

**Background:**

Increasing evidence suggests that maternal distress is a risk factor for development of respiratory infections and allergic diseases in the offspring. We aim to evaluate the link between maternal distress during critical periods in early life, namely the preconception, pregnancy and postnatal periods, and development of respiratory infections and allergic diseases in the offspring from the Singapore PREconception Study of long Term maternal and child Outcomes (S-PRESTO) cohort.

**Methods:**

Maternal perceived distress was evaluated using validated questionnaires including Beck Depression Inventory-II (BDI-II) administered during three time periods: preconception (three months apart at four timepoints), pregnancy (during each trimester) and postnatal (3 and 6 months post-delivery). Child eczema, rhinitis and wheeze outcomes were evaluated using a modified ISAAC questionnaire at ages 3, 6, 12, and 18 months. Child allergic sensitization was determined by skin prick testing at 18 months.

**Results:**

Among 332 mother-child pairs studied, higher maternal distress during preconception and pregnancy increased the risks of wheeze development in the first 18 months; for example, preconception and pregnancy BDI-II scores ≥20 were associated with increased risks of wheeze by 18 months [adjusted risk ratios 3.2 (95%CI 1.1–9.4) and 2.5 (1.0–5.9), respectively]. Emotional and practical support from family during preconception decreased the risks of offspring wheeze. No associations were observed between maternal distress and offspring eczema, rhinitis and allergic sensitization.

**Conclusion:**

Maternal distress during critical early life periods was associated with offspring wheeze in the first 18 months of life. Supporting maternal mental health even before pregnancy could reduce the risk of offspring wheeze.

## Graphical Abstract

Maternal distress during preconception and pregnancy periods increased the risk of wheeze development in children while social support decreased the risk. No association was observed between maternal distress experienced during all three window periods and eczema, rhinitis and allergic sensitization in the offspring. 
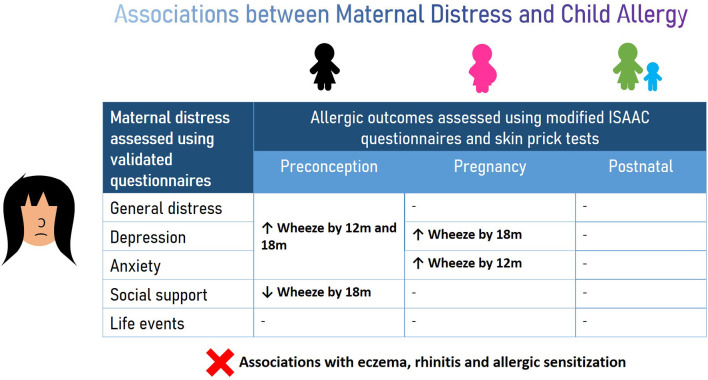

## Introduction

Allergy and respiratory infections are global health issues (1, 2) and impact the quality of life as well as school performance of children. The rapid increase in prevalence of allergic diseases and respiratory infections is postulated to be due to environmental and lifestyle factors such as psychosocial distress, which is defined as an emotional state of discomfort resulting from exposure to stress (3). Findings from epidemiological studies strongly suggest that maternal health during preconception and over the course of pregnancy and postnatal development influence child's health. The influence of early life environment on child's health forms the basis for the Developmental Origins of Health and Disease (DOHaD) paradigm which hypothesizes that early environmental stimuli during preconception, pregnancy and early life may influence fetal and neonatal immune development and cause development of diseases including eczema, asthma, allergic rhinitis and allergic sensitization during early life (4–6).

Increasing evidence suggests that maternal distress is a risk factor for development of allergic and respiratory diseases in the offspring. In a meta-analysis of 30 studies and a cross-sectional study involving 3,758 Italian mother-child pairs, prenatal maternal distress was associated with increased risk of development of eczema, rhinitis, wheeze, and asthma in the offspring (7, 8). Prenatal maternal anxiety, depression and distress were also associated with higher risk of eczema in two Korean cohorts of children at 4 and 5 years of age (9). The Generation R study from the Netherlands reported that mothers with higher distress levels had an increased risk of having offspring who wheezed at 1–4 years of age (10). In China, schoolchildren had increased risk of rhinitis if their mothers experienced symptoms of depression during and after pregnancy (11). Furthermore, the prevalence of maternal distress has increased in recent years; prenatal depression was twice as common in a cohort of young mothers as compared to their mothers, while severe postnatal depression increased by 34% over a five-year period in a US study (12, 13).

**Figure 1:**
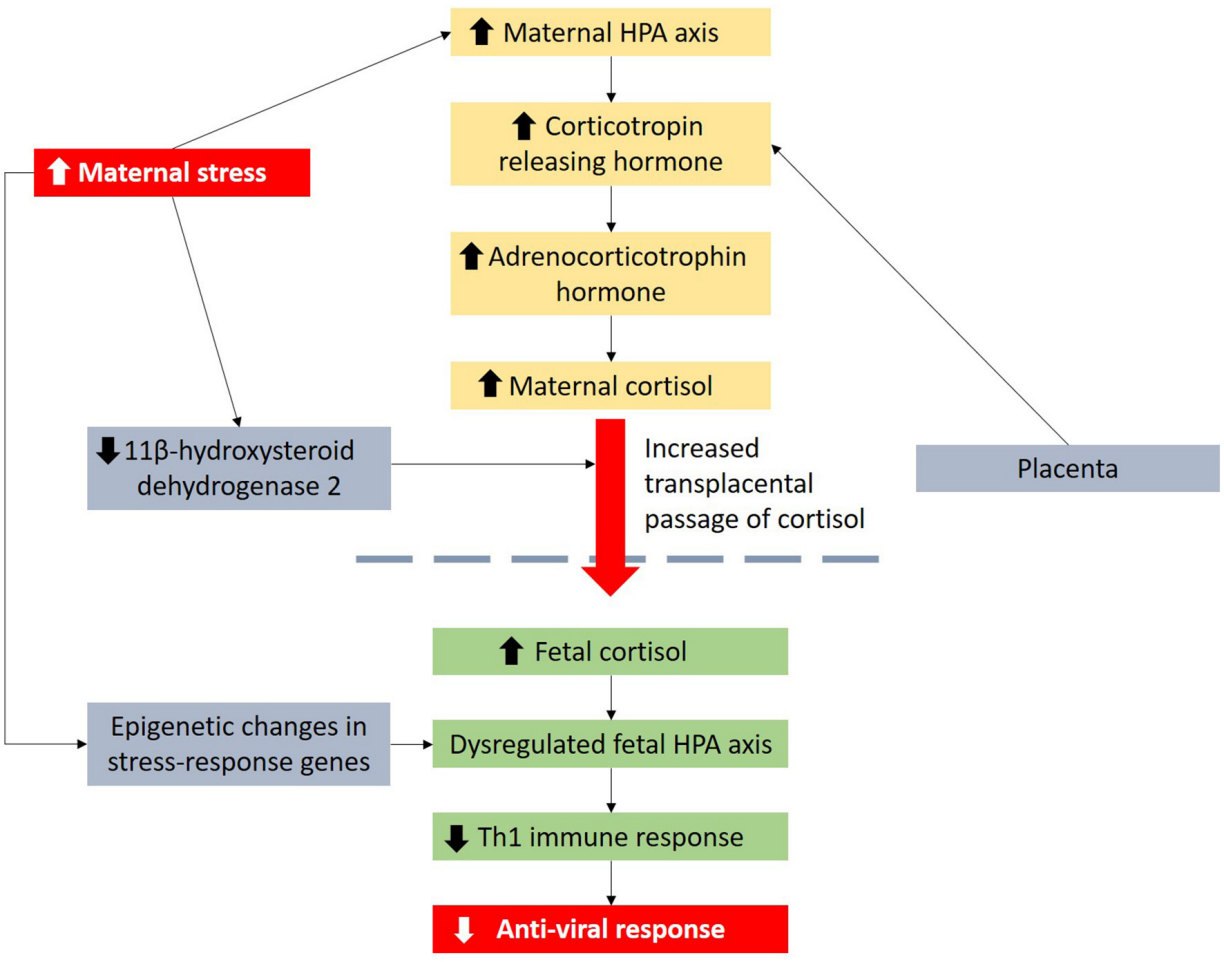
Maternal distress is linked to viral infection in children through dysregulation of the HPA axis and influence of Th1 immune response. Maternal distress can also lead to epigenetic changes in stress-response genes.

Extensive research over the past years showed that maternal distress can influence the offspring's immune system by regulating the hypothalamic-pituitary-adrenal (HPA) axis that plays a pivotal role in regulating adaptive immunological responses to stressors (Figure 1). High maternal distress promotes cortisol production and secretion, downregulates expression of 11β-hydroxysteroid dehydrogenase 2 in the placenta and consequently exposes the fetus to excessive cortisol levels (14–16). Elevated cortisol exposure is linked to dysregulated HPA axis function in infants, which can aggravate allergic inflammation (17, 18) and favor a T-helper 2 (Th2) immune response by inhibiting interleukin-12, a Th1 cytokine (19, 20). Studies have also reported prenatal maternal distress to be linked to higher respiratory infection rates/risk in the offspring possibly due to dysregulated HPA axis and poorer maternal dietary and lifestyle habits (21, 22).

While several studies have focused on prenatal maternal distress (23), very few studies have explored the association between maternal distress during preconception and allergy as well as respiratory infections in the offspring. Among 3,008 mother-child pairs in the Southampton Women's Survey, a positive association was found between preconception maternal distress and development of eczema in infants at 12 months (24). A Swedish study of 3.2 million mother-child pairs showed that the offspring of mothers who experienced severe life events up to 6 months before and during pregnancy had increased risk of hospitalization due to asthma and other related diagnoses including bronchiolitis, eczema and respiratory infections especially in the first 2 years of life (25). Our focus on early life starting periconceptually and across critical development periods allows us to examine the earliest possible developmental influences independent of numerous confounders that emerge subsequently. This will enable the identification of earliest risk factors where interventions may be more effective.

To the best of our knowledge, there are no studies that have evaluated the impact of maternal distress during all three critical time periods, namely preconception, pregnancy and postnatal, and the development of allergic diseases and respiratory infections in the offspring. Hence, we aimed to evaluate this relationship in the Singapore PREconception Study of long Term maternal and child Outcomes (S-PRESTO) cohort.

## Materials and Methods

### S-PRESTO Study Design and Definition of Allergic Outcomes

The S-PRESTO study is a prospective cohort study which recruited women aged 18–45 years old who planned to conceive and deliver in Singapore, out of which 373 infants were born. The detailed methodology was described by Loo et al. (26). Trained interviewers gathered information on demographic characteristics, family history of allergy, socioeconomic data, and lifestyle factors. The ISAAC modified questionnaire was used to evaluate offspring eczema, wheeze, and rhinitis symptoms at ages 3, 6, 12, and 18 months. Eczema was determined as maternally reported doctor diagnosis of eczema. Wheeze with use of nebulizer/inhaler was defined by positive responses to the questions: “Has your child ever wheezed?” and “Has your child ever been prescribed with nebulizer/inhaler treatment?”. Rhinitis was defined as a positive response to the question “Has your child had running nose, blocked or congested nose, snoring or noisy breathing during sleep or when awake that has lasted for 2 or more weeks duration?”. Cumulative eczema, wheeze with the use of nebulizer/inhaler or rhinitis by 6, 12, and 18 months were classified as “yes” when a subject answered “yes” by the time point and “no” if the subject answered “no” at all time points. Ethical approval was obtained from the SingHealth Centralised Institutional Review Board (reference 2014/692/D). This study has been registered at ClinicalTrials.gov (NCT 03531658). Written informed consent was provided by the participants.

### Allergen Sensitization

Skin prick testing (SPT) was performed at 18 months for the major relevant allergens cow's milk, whole egg, peanut, soy, wheat, shrimp, crab, and house dust mites *Dermatophagoides pteronyssinus* (*Der p*), *Dermatophagoides farina* (*Derp f* ) and *Blomia tropicalis* (*Blo t*). The infant was classified as having positive SPT if any of the SPT to the allergens was positive (minimum wheal size of 3 mm) and negative if all of the SPT to the allergens were negative.

### Distress Assessment

Maternal perceived distress was assessed using a battery of validated questionnaires assessing symptoms of depression [Edinburgh Postnatal Depression Scale (EPDS) and Beck Depression Inventory-II (BDI-II)], anxiety [State-Trait Anxiety Inventory (STAI) and Pregnancy Anxiety Questionnaire (PAQ)], facets of social support [Multidimensional Scale of Perceived Social Support (MSPSS)], life events [Life Experiences Survey (LES)] and levels of general perceived stress [General Health Questionnaire (GHQ), Pregnancy Experience Scale (PES) and Perceived Stress Scale (PSS)]. Depression refers to prolonged feelings of loss of interest, sadness and hopelessness (27). Anxiety refers to feelings of uneasiness or apprehension due to anticipation of future negative events (28). The MSPSS evaluates perceived support from spouse, family and friends in terms of ability to share joys and sorrows, obtain comfort, share problems and help in decision-making and solving problems (29). The questionnaires were administered at different time points from preconception to postnatal: at each trimester during pregnancy and at two time points during the postnatal period. The maximum distress during preconception, pregnancy and postnatal were computed from each of the questionnaires.

### Statistical Analysis

All analyses were performed using SPSS for Windows version 26.0 (SPSS Inc., Chicago, IL, USA). Statistical significance was set at two-sided *p* < 0.05. Descriptive statistics for numerical variables were presented as mean (SD) when normality and homogeneity assumptions were satisfied, otherwise median (IQR) were presented and *n* (%) for categorical variables. Predictors for offspring allergic outcomes by ages 6, 12, and 18 months and SPT at month 18 were assessed using modified Poisson regression for prospective studies with binary outcomes (30–33), adjusting for demographic and relevant covariates period of maximum distress (if distress accessed at several time points), ethnicity, maternal age at birth, length of education, parity, smoking during pregnancy, maternal history of allergy, infant sex and gestational age at birth as assessed from literature review (7, 34). Smoking during pregnancy was not adjusted for in the postnatal period. Type 1 error for multiple comparisons were adjusted using Benjamini–Hochberg procedure with false discovery rate at 0.45.

## Results

### Study Population Characteristics

In this study, 332 mother-child pairs with data on both maternal distress and child respiratory infections and allergic outcomes were included. The mothers' mean age at delivery was 31.6 years (SD 3.2, Table 1). The majority of mothers were of Chinese ethnicity [254 (76.5%)], had at least 12 years of education [309 (93.1%)], had a history of allergy [235 (70.8%)], were nulliparous [203 (61.3%)] and did not smoke during pregnancy [310 (99.4%)]. Of the 332 infants, 182 (55.0%) were boys. There were 51 (17.1%), 73 (25.7%) and 87 (31.5%) infants who developed eczema by ages 6, 12, and 18 months, respectively. There were 106 (34.5%), 142 (48.0%) and 159 (55.2%) infants who developed rhinitis by ages 6, 12, and 18 months, respectively and 10 (3.3%), 30 (10.8%) and 33 (12.8%) wheezed and used nebulizer by ages 6, 12, and 18 months respectively. At age 18 months, 40 (16.5%) had a positive SPT.

**Table 1:** Characteristics of the study population.

	***n***	**Median (IQR), Mean (SD) or *n* (%)**
Ethnicity	332	
Chinese		254(76.5%)
Malay		47(14.2%)
Mix		13(3.9%)
Indian		18(5.4%)
Education	332	
≥12 years of education		309(93.1%)
<12 years of education		23(6.9%)
Maternal age	332	31.6 ± 3.2
Maternal allergy	332	
Yes		235(70.8%)
No		97(29.2%)
Parity	331	
Parous		128(38.7%)
Nulliparous		203(61.3%)
Smoking during pregnancy	312	
Yes		2(0.6%)
No		310(99.4%)
Infant Sex	331	
Male		182(55.0%)
Female		149(45.0%)
Gestational age at birth	329	39.0 (38.2–39.7)
Prepregnancy BMI	329	21.7 (20.1–24.6)
**Preconception**		
**General distress**		
GHQ	183	10.0 (7.0–12.0)
PSS	181	15.4 ± 5.6
**Depression**		
BDI	178	6.0 (2.0–12.0)
Period of maximum BDI (month)	178	0 (0–0)
EPDS	219	8.0 (5.0–11.0)
Period of maximum EPDS (month)	219	0 (0–0)
**Anxiety**		
STAI state	218	35.0 (26.0–40.0)
Period of maximum STAI state (month)	218	0 (0–0)
STAI trait	219	38.2 ± 8.5
Period of maximum STAI trait (month)	219	0 (0–0)
**Social support**		
MSPSS emotional support from partner	182	5.0 (4.0– 5.0)
MSPSS emotional support from family	182	4.0 (3.5–5.0)
MSPSS emotional support from friend	182	4.0 (3.5–5.0)
MSPSS practical support from family	182	4.0 (3.5–5.0)
MSPSS practical support from friend	182	4.0 (3.0–4.5)
**Life event**		
LES positive	174	7.0 (3.0–10.0)
LES negative	174	3.0 (1.0–6.0)
**Pregnancy**		
**General distress**		
PES Hassles/Uplifts frequency ratio	293	1.00 (0.90–1.25)
Period of maximum PES Hassles/Uplifts frequency ratio (week)	293	26.0 (8.0–36.0)
PES Hassles/Uplifts frequency ratio	293	1.00 (0.85–1.20)
Period of maximum PES Hassles/Uplifts intensity ratio (week)	293	26.0 (8.0–36.0)
PSS	292	17.3 ± 5.6
Period of maximum PSS (week)	292	26.0 (8.0–36.0)
**Depression**		
BDI	311	10.0 (6.0–15.0)
Period of maximum BDI (week)	311	26.0 (8.0–36.0)
EPDS	312	8.0 (5.0–11.0)
Period of maximum EPDS (week)	312	26.0 (8.0–36.0)
**Anxiety**		
STAI state	313	39.2 ± 10.9
Period of maximum STAI state (week)	313	26.0 (8.0–36.0)
STAI trait	313	39.9 ± 9.5
Period of maximum STAI trait (week)	313	26.0 (8.0–36.0)
PAQ	289	2.9 ± 0.6
Period of maximum PAQ (week)	289	26.0 (8.0–36.0)
**Social support**		
MSPSS emotional support from partner	290	5.0 (4.3–5.0)
Period of maximum MSPSS emotional support from partner (week)	290	36.0 (26.0–36.0)
MSPSS emotional support from family	290	5.0 (4.0–5.0)
Period of maximum MSPSS emotional support from family (week)	290	26.0 (26.0–36.0)
MSPSS emotional support from friend	290	4.5 (4.0–5.0)
Period of maximum MSPSS emotional support from friend (week)	290	26.0 (26.0–36.0)
MSPSS practical support from family	290	4.5 (4.0–5.0)
Period of maximum MSPSS practical support from family (week)	290	26.0 (26.0–36.0)
MSPSS practical support from friend	290	4.0 (3.5–5.0)
Period of maximum MSPSS practical support from friend (week)	290	26.0 (26.0–36.0)
**Postnatal**		
**General distress**		
PSS at month 6	172	15.4 ± 5.9
**Depression**		
BDI (included month 6)	250	9.0 (4.0–14.0)
Period of maximum BDI (month)	250	3.0 (3.0–6.0)
BDI (excluded month 6)	211	7.0 (4.0–12.0)
EPDS (included month 6)	250	7.0 (3.0–10.0)
Period of maximum EPDS (month)	250	6.0 (3.0–6.0)
EPDS (excluded month 6)	211	5.0 (2.0–9.0)
**Anxiety**		
STAI state (included month 6)	249	35.0 (29.5–44.0)
Period of maximum STAI state(month)	249	3.0 (3.0–6.0)
STAI state (excluded month 6)	211	33.0 (25.0–41.0)
STAI trait (included month 6)	249	38.0 (31.0–46.0)
Period of maximum STAI trait (month)	249	3.0 (3.0–6.0)
STAI trait (excluded month 6)	211	36.0 (28.0–43.0)
**Social support**		
MSPSS emotional support from partner at month 6	179	4.7 (4.0–5.0)
MSPSS emotional support from family at month 6	179	4.0 (3.5–5.0)
MSPSS emotional support from friend at month 6	178	4.0 (3.5–5.0)
MSPSS practical support from family at month 6	179	4.0 (3.5–5.0)
MSPSS practical support from friend at month 6	179	4.0 (3.0–4.5)
**Life event**		
LES positive at month 6	173	6.0 (3.0–10.0)
LES negative at month 6	173	5.0 (2.0–9.0)
Eczema by 6 months	299	
Yes		51 (17.1%)
No		248 (82.9%)
Eczema by 12 months	284	
Yes		73 (25.7%)
No		211 (74.3%)
Eczema by 18 months	276	
Yes		87 (31.5%)
No		189 (68.5%)
Rhinitis by 6 months	307	
Yes		106 (34.5%)
No		201 (65.5%)
Rhinitis by 12 months	296	
Yes		142 (48.0%)
No		154 (52.0%)
Rhinitis by 18 months	288	
Yes		159 (55.2%)
No		129 (44.8%)
Wheeze by 6 months	299	
Yes		10 (3.3%)
No		289 (96.7%)
Wheeze by 12 months	277	
Yes		30 (10.8%)
No		247 (89.2%)
Wheeze by 18 months	258	
Yes		33 (12.8%)
No		225 (87.2%)
Month 18 SPT	242	
Positive		40 (16.5%)
Negative		202 (83.5%)

*IQR, Interquartile range; SD, standard deviation; BDI-II, Beck Depression Inventory-II; EPDS, Edinburgh Postnatal Depression Scale; GHQ, General Health Questionnaire; LES, Life Experiences Survey; MSPSS, Multidimensional Scale of Perceived Social Support; PAQ, Pregnancy Anxiety Questionnaire; PES, General Health Questionnaire; PSS, Perceived Stress Scale; STAI, State-Trait Anxiety Inventory*.

### Association Between Maternal Distress and Allergic Outcomes in the Offspring

#### General Distress

Univariate associations are presented in Supplementary Tables 1–4. In multivariate analyses, higher preconception GHQ scores were associated with increased risk of wheeze by 12 and 18 months after adjusting for demographic and relevant covariates (AdjRR 1.2, 95% CI 1.1–1.4 and AdjRR 1.2, 95% CI 1.1–1.3, respectively, Table 2). Higher preconception PSS scores were associated with increased risk of wheeze by 12 and 18 months (AdjRR 1.1, 95% CI 1.0–1.2 and AdjRR 1.1, 95% CI 1.0–1.2, respectively). There were no associations between GHQ and Perceived Stress Scale scores and PES Hassles/Uplifts frequency and intensity ratios with child eczema, rhinitis and allergic sensitization outcomes (Supplementary Tables 5–7).

**Table 2:** Multivariate poisson regression of wheeze by 6, 12, and 18 months.

	**6 months**			**12 months**			**18 months**		
	***n***	**RR (95% CI)**	***p*-value^b^**	***n***	**RR (95% CI)**	***p*-value^b^**	***n***	**RR (95% CI)**	***p*-value^b^**
**Preconception**									
**General distress**									
GHQ	160	1.4 (1.0–2.1)	0.08	146	1.2 (1.1–1.4)	**<0.001**	136	1.2 (1.1–1.3)	**<0.001**
PSS	159	1.0 (0.8–1.2)	0.855	145	1.1 (1.0–1.2)	**0.038**	136	1.1 (1.0–1.2)	**0.044**
PSS ≥ 14	159	0.44 (0.05–4.00)	0.469	145	1.4 (0.4–4.1)	0.593	136	1.3 (0.5–3.9)	0.588
**Depression**									
BDI	154	1.0 (0.9–1.2)	0.549	138	1.07 (1.01–1.13)	**0.026**	126	1.06 (1.00–1.12)	**0.037**
BDI	154			138			126		
0–13		1			1			1	
14–19		4.5 (0.3–70.5)	0.28		0.69 (0.06–7.97)	0.768		0.87 (0.09–8.15)	0.905
≥20		7.5 (0.5–101.7)	0.131		3.5 (1.2–10.9)	**0.026**		3.2 (1.1–9.4)	**0.032**
EPDS	189	0.95 (0.76–1.20)	0.688	170	1.1 (1.0–1.3)	**0.036**	158	1.1 (1.0–1.2)	**0.04**
EPDS ≥ 13	189	1.3 (0.1–22.0)	0.866	170	2.3 (0.7–7.3)	0.173	158	1.9 (0.6–5.9)	0.281
**Anxiety**									
STAI state	188	0.98 (0.88–1.09)	0.684	169	1.05 (0.99–1.10)	0.108	157	1.04 (0.99–1.10)	0.083
STAI trait	188	1.0 (0.9–1.2)	0.788	170	1.06 (1.00–1.13)	**0.037**	158	1.07 (1.01–1.13)	**0.016**
STAI trait ≥ 40	189	0.63 (0.08–4.89)	0.655	170	1.6 (0.6–4.5)	0.334	158	1.8 (0.7–4.6)	0.195
**Social support**									
MSPSS emotional support from partner	159	0.61 (0.10–3.68)	0.588	145	1.0 (0.6–1.9)	0.883	136	0.84 (0.53–1.33)	0.457
MSPSS emotional support from family	159	1.1 (0.2–7.1)	0.89	145	0.64 (0.40–1.02)	0.061	136	0.58 (0.38–0.89)	**0.012**
MSPSS emotional support from friend	159	0.99 (0.13–7.34)	0.992	145	1.1 (0.6–1.9)	0.745	136	0.86 (0.51–1.45)	0.575
MSPSS practical support from family	159	1.0 (0.2–4.4)	0.964	145	0.67 (0.42–1.06)	0.085	136	0.66 (0.43–0.99)	**0.047**
MSPSS practical support from friend	159	1.4 (0.2–8.2)	0.69	145	1.2 (0.6–2.2)	0.623	136	1.0 (0.6–1.7)	0.973
**Life event**									
LES positive	151	1.2 (1.0–1.4)	0.117	136	1.0 (0.9–1.1)	0.847	127	0.99 (0.89–1.09)	0.795
LES negative	151	1.1 (0.9–1.4)	0.257	136	1.1 (1.0–1.2)	0.101	127	1.1 (1.0–1.2)	0.243
**Pregnancy**									
**General distress**									
PES Hassles/Uplifts frequency ratio	252	0.75 (0.19–3.02)	0.686	235	1.2 (0.8–1.9)	0.445	221	1.2 (0.7–1.8)	0.525
PES Hassles/Uplifts intensity ratio	252	1.4 (0.2–11.1)	0.736	235	1.3 (0.4–4.5)	0.623	221	1.3 (0.4–4.1)	0.634
PSS	251	1.1 (1.0–1.2)	0.177	234	1.06 (0.99–1.14)	0.071	220	1.06 (0.99–1.13)	0.083
PSS ≥ 27	251	2.6 (0.2–35.5)	0.479	234	2.8 (0.8–10.4)	0.119	220	2.4 (0.6–8.7)	0.196
**Depression**									
BDI	266	1.0 (0.9–1.1)	0.637	246	1.04 (0.99–1.08)	0.116	231	1.03 (0.99–1.08)	0.156
BDI	266			246			231		
0–13		1			1			1	
14–19		3.1 (0.5–20.6)	0.246		1.5 (0.5–4.2)	0.49		1.4 (0.5–3.9)	0.561
≥20		1.3 (0.1–11.8)	0.807		2.3 (0.9–5.8)	0.071		2.5 (1.0–5.9)	**0.042**
EPDS	266	1.0 (0.9–1.2)	0.649	246	1.1 (1.0–1.2)	0.098	231	1.06 (0.99–1.14)	0.115
EPDS ≥ 13	266	NA	NA	246	1.1 (0.4–2.7)	0.912	231	1.1 (0.5–2.7)	0.786
**Anxiety**									
STAI state	267	1.02 (0.95–1.09)	0.568	247	1.04 (1.00–1.08)	**0.03**	232	1.03 (1.00–1.06)	0.087
STAI trait	267	1.0 (0.9–1.1)	0.888	247	1.03 (0.99–1.07)	0.211	232	1.02 (0.98–1.06)	0.309
STAI trait ≥ 40	267	2.3 (0.4–12.4)	0.32	247	2.3 (1.0–5.2)	0.056	232	1.9 (0.9–4.1)	0.104
PAQ	248	1.2 (0.3–4.5)	0.827	231	1.6 (0.8–3.0)	0.183	218	1.4 (0.8–2.6)	0.247
**Social support**									
MSPSS emotional support from partner	249	0.94 (0.19–4.70)	0.937	232	0.93 (0.43–2.01)	0.854	219	0.95 (0.43–2.09)	0.895
MSPSS emotional support from family	249	1.1 (0.4–2.9)	0.886	232	0.96 (0.58–1.59)	0.878	219	0.88 (0.55–1.41)	0.586
MSPSS emotional support from friend	249	0.76 (0.32–1.83)	0.538	232	1.1 (0.7–1.9)	0.61	219	1.08 (0.67–1.73)	0.754
MSPSS practical support from family	249	0.65 (0.27–1.53)	0.323	232	0.76 (0.47–1.20)	0.238	219	0.71 (0.45–1.12)	0.144
MSPSS practical support from friend	249	0.66 (0.29–1.50)	0.319	232	1.1 (0.7–1.8)	0.698	219	1.1 (0.7–1.8)	0.661
**Postnatal^c^**									
**General distress**									
PSS	NA	NA	NA	154	1.04 (0.95–1.15)	0.359	136	1.0 (0.9–1.1)	0.484
PSS	NA	NA	NA	154			143		
0–13					1			1	
14–26					0.43 (0.12–1.57)	0.204		0.73 (0.24–2.21)	0.58
≥27					3.1 (0.5–18.0)	0.205		2.8 (0.5–16.0)	0.247
**Depression**									
BDI	201^a^	1.1 (0.9–1.2)^a^	0.394^a^	219	1.02 (0.98–1.07)	0.302	203	1.02 (0.98–1.06)	0.415
BDI ≥ 14	201^a^	4.0 (0.3–54.2)^a^	0.297^a^	219	1.4 (0.5–3.9)	0.521	203	1.2 (0.4–3.4)	0.697
EPDS	201^a^	1.1 (0.9–1.3)^a^	0.170^a^	219	0.99 (0.91–1.08)	0.873	203	0.98 (0.90–1.06)	0.588
EPDS ≥ 13	201^a^	3.8 (0.3–45.3)^a^	0.292^a^	219	1.1 (0.4–3.5)	0.816	193	1.0 (0.3–3.1)	0.957
**Anxiety**									
STAI state	201^a^	1.02 (0.95–1.10)^a^	0.507^a^	218	1.03 (1.00–1.07)	0.067	202	1.02 (0.99–1.06)	0.162
STAI trait	201^a^	1.0 (0.9–1.1)^a^	0.527^a^	218	1.02 (0.98–1.06)	0.264	202	1.02 (0.98–1.06)	0.244
STAI trait ≥ 40	201^a^	2.9 (0.5–16.9)^a^	0.226^a^	218	1.2 (0.5–2.7)	0.687	202	1.3 (0.6–2.8)	0.519
**Social support**									
MSPSS emotional support from partner	NA	NA	NA	163	0.87 (0.5–1.52)	0.622	152	0.91 (0.52–1.59)	0.745
MSPSS emotional support from family	NA	NA	NA	163	0.90 (0.55–1.49)	0.691	152	0.88 (0.55–1.40)	0.579
MSPSS emotional support from friend	NA	NA	NA	163	1.2 (0.7–2.1)	0.543	152	1.1 (0.6–1.8)	0.844
MSPSS practical support from family	NA	NA	NA	163	0.73 (0.43–1.25)	0.255	152	0.74 (0.45–1.22)	0.233
MSPSS practical support from friend	NA	NA	NA	163	1.0 (0.6–1.8)	0.941	152	1.1 (0.6–1.8)	0.766
**Life event**									
LES positive	NA	NA	NA	158	1.0 (0.9–1.1)	0.957	149	1.0 (0.9–1.1)	0.927
LES negative	NA	NA	NA	158	1.04 (0.97–1.12)	0.305	149	1.02 (0.95–1.10)	0.564

*BDI-II, Beck Depression Inventory-II; EPDS, Edinburgh Postnatal Depression Scale; GHQ, General Health Questionnaire; LES, Life Experiences Survey; MSPSS, Multidimensional Scale of Perceived Social Support; PAQ, Pregnancy Anxiety Questionnaire; PES, General Health Questionnaire; PSS, Perceived Stress Scale; STAI, State-Trait Anxiety Inventory. CI, confidence interval; RR, risk ratio*.

*RR = 1.0 is the reference category*.

*NA, not applicable (0 count/stress accessed at the same time point with the outcome)*.

a *Month 3 postnatal stress*.

b *Adjusted for period of maximum stress (if stress accessed at several time points), ethnicity, maternal age at birth, length of education, parity, smoking during pregnancy, maternal history of allergy, infant sex and gestational age at birth*.

c *Adjusted for period of maximum stress (if stress accessed at several time points), ethnicity, maternal age at birth, length of education, parity, maternal history of allergy, infant sex and gestational age at birth*.

*Significant p-value after Benjamini-Hochberg correction with false discovery rate at 0.45 and n = 148 in bold*.

#### Depression

Higher preconception BDI scores were associated with increased risk of wheeze by 12 and 18 months (AdjRR 1.07, 95% CI 1.01–1.13 and AdjRR 1.06, 95% CI 1.00–1.12, respectively, Table 2). Further analysis with BDI categories showed that preconception BDI scores ≥ 20 increased the risk of wheeze by 12 months (AdjRR 3.5, 95% CI 1.2–10.9). Preconception and pregnancy BDI scores ≥ 20 were associated with increased risk of wheeze by 18 months (AdjRR 3.2, 95% CI 1.1–9.4, respectively, AdjRR 2.5, 95% CI 1.0–5.9, respectively).

Higher preconception EPDS scores were associated with increased risk of wheeze by 12 and 18 months (AdjRR 1.1, 95% CI 1.0–1.3 and AdjRR 1.1, 95% CI 1.0–1.2, respectively).

There were no associations between BDI and EPDS scores and child eczema, rhinitis and allergic sensitization outcomes (Supplementary Tables 5–7).

#### Anxiety

Higher preconception STAI trait scores were associated with increased risk of wheeze by 12 and 18 months, respectively (AdjRR 1.06, 95% CI 1.00–1.13 and AdjRR 1.07, 95% CI 1.01–1.13, respectively, Table 2). Higher pregnancy STAI state scores were associated with increased risk of wheeze by 12 months (AdjRR 1.04, 95% CI 1.00–1.08).

There were no associations between STAI trait and state scores and Pregnancy Anxiety Questionnaire scores and child eczema, rhinitis and allergic sensitization outcomes (Supplementary Tables 5–7).

#### Social Support

Higher preconception MSPSS emotional support from family scores and practical support from family scores were associated with a lower risk of wheeze by age 18 months (AdjRR 0.58, 95% CI 0.38–0.89) and AdjRR 0.66, 95% CI 0.43–0.99, respectively, Table 2).

There were no associations between MSPSS emotional and physical support scores and child eczema, rhinitis and allergic sensitization outcomes (Supplementary Tables 5–7).

#### Life Events

There were no associations between positive and negative LES scores and child eczema, rhinitis, wheeze with the use of nebuliser and allergic sensitization outcomes (Supplementary Tables 5–7).

## Discussion

In this study, we examined aspects of maternal distress during preconception, pregnancy and postnatal periods using a battery of validated questionnaires to assess maternal distress and their associations with eczema, rhinitis, wheeze, and allergic sensitization outcomes in the offspring in early life.

We observed associations of higher maternal distress during preconception and pregnancy with higher risks of wheeze development by ages 12 and 18 months, while social support decreased the risk. Supportive evidence is provided by the GUSTO cohort from Singapore which reported significant associations between maternal depression during pregnancy and child wheeze by age 1 year (35) and a meta-analysis which reported a 56% higher risk of wheeze in offspring whose mothers experienced prenatal psychological distress levels (36), suggesting that control of maternal distress through social support can reduce offspring wheeze risk.

Wheezing illnesses are mainly caused by respiratory viruses, and not by allergy, in young children (37). Supporting evidence of the role of viruses in the etiology of wheeze has been provided by a number of studies. The COAST study in the US identified 90% of wheezing in children up to 3 years of age to be associated with viral etiology (37). A US study of children who visited the emergency department for wheezing reported that respiratory viruses were detected in 82% of wheezing infants younger than age 2 years (38). We postulate that the associations between maternal distress and wheeze may be due to lower anti-viral responses in the offspring (Figure 1). Hyper-reactivity of the HPA axis to stress is linked to enhanced production of glucocorticoids which inhibit Th1 responses that are essential in anti-viral responses (39, 40). Maternal distress during preconception and pregnancy can also result in persisting and epigenetic changes in genes involved in stress responses (41, 42) which may be passed to the offspring. For example, murine models showed that maternal preconception distress resulted in increased expression of corticotropin releasing factor type 1, a protein key in stress responses, in mature oocytes and offspring brain (43). Higher cord blood Nuclear Receptor Subfamily 3 Group C Member 1 (NR3C1) CpG3 methylation is also linked to higher maternal depression and anxiety during third trimester of pregnancy and increased infant salivary cortisol stress responses at 3 months of age, suggesting increased HPA stress response in infants (44).

Supporting evidence of the link between maternal distress and lower immunity in the offspring is also provided by a number of studies; Rusconi et al. reported that higher maternal GHQ scores i.e. poorer mental health during and after pregnancy increased the risk of wheezing as well as respiratory and gastroenteric infections in the offspring at 1–2 years (45). In another cohort of more than 1.6 million Danish children, maternal stressful events up to 11 months before pregnancy were linked to higher risk of infectious disease hospitalization in the offspring (46).

In this study, we did not observe any associations between maternal distress experienced preconception, or during the pregnancy or postnatal periods and eczema, rhinitis and allergic sensitization in the offspring. Existing studies have yielded conflicting results on the association between maternal distress and these allergic outcomes (47). In support of our findings, the Ulm SPATZ Health Study reported that mothers belonging to the highest quartile in relation to prenatal distress, anxiety and depression did not observe more parental report of child eczema diagnosis by 2 years (48). The GUSTO cohort reported non-significant associations between maternal depression and anxiety during pregnancy as assessed by EPDS and STAI, respectively, with child eczema by age 1 year (35). The LISA Study did not observe significant associations between maternal distress during pregnancy and child eczema in the first 6 years of life (49). Similarly, the ALSPAC study reported no associations between maternal anxiety at 18 and 32 weeks of pregnancy and child allergic sensitization at 7.5 years (34). The Western Australian Pregnancy Cohort also did not observe significant associations between maternal distress during pregnancy and child rhinitis at ages 6 and 14 years (50). Contrary to our observations, the UK Southampton Women's Survey observed that preconception distress as assessed by the Short Form (36) Health Survey was linked to higher risk of eczema development in the offspring at 12 months (24). The China National Birth Cohort Study also reported an association between maternal distress during pregnancy and infant eczema development at 6 months (51). Another study of 24200 mother-child pairs in Taiwan reported that postpartum depression at 6 months was associated with an increased risk of child eczema at 3 years (52). The Viadana study reported that maternal stressful life events during pregnancy increased the risk of allergic rhinitis in children aged ~8.5 years (8). Possible explanations for these discrepancies include the use of different types of distress assessments methods. For example, the Ulm SPATZ study used Trier Inventory of Chronic Stress, Pregnancy Related Anxiety Questionnaire and Hospital Anxiety and Depression Scale while the UK Southampton Women's Survey used the Short Form (36) Health Survey. Moreover, although rhinitis can also be viral-induced (53), our study did not differentiate between allergic and infectious rhinitis which might have reduced the strength of associations between maternal distress and rhinitis. Taken together, our observations suggest that maternal distress may result in specific lower anti-viral immune responses to respiratory viruses in the offspring rather than allergic disease development.

The strengths of this study include the comprehensive assessment of maternal distress at multiple time points via a battery of validated questionnaires from preconception to pregnancy and after birth. The specific design of this preconceptional study can offer new insights into the earliest precursors and risk factors of child's health in an Asian population. A limitation of our study is the modest sample size. However, we have increased the reliability of our results using robust statistical methods. Although we used questionnaires to gather information on allergic disease diagnosis and maternal mental health, these questionnaires had also been used by numerous studies in the field (54–71). This limitation is also mitigated by regular follow-ups to reduce recall bias. We also did not assess physiological responses to maternal distress in both mothers and offspring and this should be evaluated in future research.

In conclusion, maternal distress during critical early life periods was associated with an increased risk of wheeze development in children in the first 18 months of life. This study highlights the importance of supporting maternal mental health, even before pregnancy, to improve offspring's health.

## Data Availability Statement

The data that support the findings of this study are available on request from the corresponding author. The data are not publicly available due to privacy or ethical restrictions.

## Ethics Statement

The studies involving human participants were reviewed and approved by SingHealth Centralised Institutional Review Board (reference 2014/692/D). Written informed consent to participate in this study was provided by the participants' legal guardian/next of kin.

## Author Contributions

HL and QY analyzed the data and wrote the manuscript. MK provided intellectual input and wrote the manuscript. YHC provided statistical advice and intellectual input. ET, AG, OT, JE, KG, PG, YSC, JC, HV, BL, LS and MM contributed to the study design and provided intellectual input. EL conceptualized the study design, contributed to the analysis and wrote the manuscript. All authors critically reviewed the manuscript.

## Funding

This work is supported by the Singapore National Research Foundation under its Translational and Clinical Research (TCR) Flagship Programme, administered by the Singapore Ministry of Health's National Medical Research Council (NMRC), Singapore-NMRC/TCR/004-NUS/2008; NMRC/TCR/012-NUHS/2014. Additional funding is provided by the Singapore Institute for Clinical Sciences, Agency for Science and Technology. KG is supported by the UK Medical Research Council (MC_UU_12011/4), the National Institute for Health Research [NIHR Senior Investigator (NF-SI-0515-10042) and NIHR Southampton Biomedical Research Centre (IS-BRC-1215-20004)], the European Union (Erasmus+ Programme ImpENSA 598488-EPP-1-2018-1-DE-EPPKA2-CBHE-JP) and the British Heart Foundation (RG/15/17/3174).

## Conflict of Interest

KG has received reimbursement for speaking at conferences sponsored by Nestle. KG and YC are part of an academic consortium that has received research funding from Abbot Nutrition, Nestle and Danone. The remaining authors declare that the research was conducted in the absence of any commercial or financial relationships that could be construed as a potential conflict of interest.

## Publisher's Note

All claims expressed in this article are solely those of the authors and do not necessarily represent those of their affiliated organizations, or those of the publisher, the editors and the reviewers. Any product that may be evaluated in this article, or claim that may be made by its manufacturer, is not guaranteed or endorsed by the publisher.

## Abbreviations

BDI-II: Beck Depression Inventory-II
*Blo t*: *Blomia tropicalis*
*Der f*: *Dermatophagoides farina*
Der p: *Dermatophagoides pteronyssinus*
EPDS: Edinburgh Postnatal Depression Scale
GHQ: General Health Questionnaire
HPA: Hypothalamic-pituitary-adrenal
LES: Life Experiences Survey
MSPSS: Multidimensional Scale of Perceived Social Support
PAQ: Pregnancy Anxiety Questionnaire
PES: General Health Questionnaire
PSS: Perceived Stress Scale
S-PRESTO: Singapore PREconception Study of long Term maternal and child Outcomes
SPT: Skin prick testing
STAI: State-Trait Anxiety Inventory.
