# Supplementary material for: Associations Between Maternal Distress During Early Life Periods and Offspring Respiratory Infections and Allergic Outcomes
Source: Front Pediatr. 2022 Mar 30;10:749323. doi: 10.3389/fped.2022.749323 (PMC9006870; doi:10.3389/fped.2022.749323)
Supplement: Supplementary file 1 [file Data_Sheet_1.docx]

**Supplementary Tables**

**Supplementary Table 1.** Univariate poisson regression of eczema by 6, 12 and 18 months.

|  | 6 months | | | 12 months | | | 18 months | | |
| --- | --- | --- | --- | --- | --- | --- | --- | --- | --- |
|  | n | RR (95% CI) | p-value | n | RR (95% CI) | p-value | n | RR (95% CI) | p-value |
| Ethnicity  Chinese  Malay  Indian  Mix | 299 | 3.4(0.5 – 24.9)  1.2(0.1 – 12.0)  1.0  1.3(0.1 – 20.9) | 0.223  0.850  0.850 | 284 | 5.0(0.7 – 35.7)  3.4(0.4 – 27.2)  1.0  1.5(0.1 – 24.7) | 0.112  0.249  0.758 | 276 | 5.3(0.7 – 37.8)  4.2(0.5 – 32.8)  1.0  1.4(0.1 – 21.8) | 0.099  0.167  0.826 |
| Education ≥ 12 years | 299 | 3.8(0.5 – 27.3) | 0.188 | 284 | 1.3(0.5 – 3.6) | 0.603 | 276 | 1.1(0.5 – 2.8) | 0.770 |
| Maternal age | 299 | 1.04(0.96 – 1.13) | 0.354 | 284 | 1.02(0.95 – 1.09) | 0.578 | 276 | 1.02(0.96 – 1.09) | 0.493 |
| Maternal allergy | 299 | 2.5(1.1 – 5.5) | **0.025** | 284 | 2.3(1.2 – 4.6) | **0.012** | 276 | 1.6(0.9 – 2.7) | 0.091 |
| Parous | 298 | 0.84(0.48 – 1.50) | 0.562 | 283 | 0.84(0.52 – 1.36) | 0.490 | 275 | 0.90(0.58 – 1.39) | 0.634 |
| Male infant | 299 | 1.4(0.8 – 2.6) | 0.206 | 284 | 1.1(0.7 – 1.8) | 0.659 | 276 | 1.1(0.7 – 1.6) | 0.753 |
| Gestational age at birth | 297 | 0.94(0.79 – 1.13) | 0.516 | 282 | 0.96(0.82 – 1.13) | 0.637 | 274 | 0.91(0.80 – 1.03) | 0.139 |
| **Preconception** |  |  |  |  |  |  |  |  |  |
| General stress |  |  |  |  |  |  |  |  |  |
| GHQ | 173 | 1.02(0.96 – 1.09) | 0.454 | 163 | 1.05(0.99 – 1.10) | 0.089 | 157 | 1.03(0.97 – 1.08) | 0.338 |
| PSS | 171 | 1.01(0.95 – 1.07) | 0.677 | 161 | 1.02(0.97 – 1.07) | 0.410 | 155 | 1.01(0.96 – 1.06) | 0.635 |
| PSS >=14 | 171 | 1.1(0.5 – 2.3) | 0.756 | 161 | 1.0(0.6 – 1.9) | 0.888 | 155 | 1.0(0.6 – 1.8) | 0.904 |
| Depression |  |  |  |  |  |  |  |  |  |
| BDI | 166 | 0.97(0.92 – 1.02) | 0.211 | 154 | 1.00(0.96 – 1.04) | 0.929 | 147 | 1.00(0.96 – 1.03) | 0.920 |
| BDI  0-13  14-19  >=20 | 166 | 1.0  0.76(0.18 – 3.18)  0.41(0.10 – 1.73) | 0.706  0.227 | 154 | 1.0  1.2(0.4 – 3.3)  0.89(0.38 – 2.11) | 0.742  0.795 | 147 | 1.0  1.1(0.4 – 3.0)  0.95(0.43 – 2.12) | 0.871  0.905 |
| Period of maximum BDI | 166 | 0.97(0.77 – 1.22) | 0.804 | 154 | 1.1(0.9 – 1.2) | 0.349 | 147 | 1.0(0.9 – 1.2) | 0.551 |
| EPDS | 204 | 0.95(0.88 – 1.03) | 0.205 | 190 | 1.0(0.9 – 1.1) | 0.919 | 183 | 0.99(0.94 – 1.06) | 0.857 |
| EPDS>=13 | 204 | 0.98(0.38 – 2.50) | 0.963 | 190 | 1.2(0.6 – 2.6) | 0.563 | 183 | 1.4(0.7 – 2.7) | 0.377 |
| Period of maximum EPDS | 204 | 1.1(1.0 – 1.3) | 0.134 | 190 | 1.1(1.0 – 1.3) | 0.110 | 183 | 1.1(0.9 – 1.3) | 0.262 |
| Anxiety |  |  |  |  |  |  |  |  |  |
| STAI state | 203 | 0.98(0.95 – 1.02) | 0.292 | 189 | 1.00(0.97 – 1.02) | 0.761 | 182 | 0.99(0.96 – 1.02) | 0.540 |
| Period of maximum STAI state | 203 | 1.0(0.9 – 1.2) | 0.990 | 189 | 1.0(0.9 – 1.2) | 0.485 | 182 | 1.1(0.9 – 1.2) | 0.361 |
| STAI trait | 204 | 0.98(0.95 – 1.02) | 0.355 | 190 | 1.00(0.97 – 1.03) | 0.972 | 183 | 0.99(0.96 – 1.02) | 0.671 |
| STAI trait >=40 | 204 | 0.74(0.39 – 1.41) | 0.361 | 190 | 0.84(0.49 – 1.43) | 0.519 | 183 | 0.79(0.47 – 1.32) | 0.365 |
| Period of maximum STAI trait | 204 | 1.0(0.9 – 1.2) | 0.887 | 190 | 1.0(0.9 – 1.2) | 0.527 | 183 | 1.0(0.9 – 1.2) | 0.417 |
| Social support |  |  |  |  |  |  |  |  |  |
| MSPSS emotional support from partner | 172 | 1.3(0.8 – 2.2) | 0.288 | 162 | 1.1(0.7 – 1.5) | 0.775 | 156 | 1.1(0.8 – 1.5) | 0.624 |
| MSPSS emotional support from family | 172 | 1.0(0.7 – 1.4) | 0.941 | 162 | 0.89(0.69 – 1.14) | 0.355 | 156 | 0.95(0.74 – 1.22) | 0.680 |
| MSPSS emotional support from friend | 172 | 1.1(0.8 – 1.6) | 0.584 | 162 | 1.1(0.8 – 1.4) | 0.635 | 156 | 1.1(0.8 – 1.4) | 0.598 |
| MSPSS practical support from family | 172 | 1.1(0.8 – 1.5) | 0.567 | 162 | 0.98(0.76 – 1.27) | 0.889 | 156 | 1.0(0.8 – 1.3) | 0.798 |
| MSPSS practical support from friend | 172 | 1.2(0.8 – 1.6) | 0.415 | 162 | 1.2(0.9 – 1.6) | 0.280 | 156 | 1.2(0.9 – 1.6) | 0.268 |
| Life event |  |  |  |  |  |  |  |  |  |
| LES positive | 163 | 0.98(0.92 – 1.04) | 0.500 | 152 | 0.97(0.92 – 1.02) | 0.282 | 146 | 0.99(0.94 – 1.03) | 0.540 |
| LES negative | 163 | 0.87(0.78 – 0.97) | **0.010** | 152 | 0.95(0.89 – 1.02) | 0.162 | 146 | 0.96(0.90 – 1.02) | 0.162 |
| **Pregnancy** |  |  |  |  |  |  |  |  |  |
| General stress |  |  |  |  |  |  |  |  |  |
| PES Hassles/Uplifts frequency ratio | 264 | 0.63(0.33 – 1.22) | 0.174 | 253 | 0.79(0.49 – 1.25) | 0.311 | 246 | 0.84(0.57 – 1.25) | 0.399 |
| Period of maximum PES Hassles/Uplifts frequency ratio | 264 | 1.00(0.98 – 1.03) | 0.783 | 253 | 1.00(0.98 – 1.02) | 0.890 | 246 | 1.00(0.98 – 1.02) | 0.803 |
| PES Hassles/Uplifts intensity ratio | 264 | 0.89(0.34 – 2.33) | 0.806 | 253 | 0.63(0.27 – 1.46) | 0.279 | 246 | 0.63(0.29 – 1.36) | 0.242 |
| Period of maximum PES Hassles/Uplifts intensity ratio | 264 | 0.99(0.96 – 1.01) | 0.310 | 253 | 0.99(0.97 – 1.01) | 0.444 | 246 | 1.00(0.98 – 1.02) | 0.864 |
| PSS | 263 | 1.03(0.98 – 1.09) | 0.205 | 252 | 1.03(0.98 – 1.07) | 0.217 | 245 | 1.01(0.97 – 1.05) | 0.808 |
| PSS  0-13  14-26  >=27 | 263 | 1.0  1.1(0.5 – 2.3)  2.2(0.7 – 7.2) | 0.835  0.184 | 252 | 1.0  1.1(0.6 – 2.1)  1.6(0.5 – 4.9) | 0.667  0.415 | 245 | 1.0  0.96(0.56 – 1.63)  1.1(0.4 – 3.3) | 0.868  0.849 |
| Period of maximum PSS | 263 | 0.99(0.97 – 1.02) | 0.665 | 252 | 0.99(0.97 – 1.01) | 0.270 | 245 | 0.99(0.98 – 1.01) | 0.587 |
| Depression |  |  |  |  |  |  |  |  |  |
| BDI | 279 | 1.00(0.97 – 1.04) | 0.819 | 266 | 1.00(0.97 – 1.03) | 0.944 | 259 | 1.00(0.97 – 1.03) | 0.843 |
| BDI  0-13  14-19  >=20 | 279 | 1.0  1.1(0.5 – 2.3)  0.87(0.36 – 2.08) | 0.776  0.748 | 266 | 1.0  0.92(0.48 – 1.77)  0.96(0.48 – 1.89) | 0.793  0.901 | 259 | 1.0  1.1(0.6 – 1.9)  0.95(0.50 – 1.82) | 0.706  0.880 |
| Period of maximum BDI | 279 | 1.00(0.97 – 1.02) | 0.847 | 266 | 1.00(0.98 – 1.02) | 0.921 | 259 | 1.00(0.98 – 1.02) | 0.921 |
| EPDS | 280 | 1.02(0.97 – 1.09) | 0.417 | 267 | 1.01(0.96 – 1.06) | 0.719 | 260 | 1.00(0.96 – 1.05) | 0.978 |
| EPDS>=13 | 280 | 1.1(0.5 – 2.3) | 0.805 | 267 | 0.83(0.44 – 1.59) | 0.577 | 260 | 0.69(0.37 – 1.31) | 0.260 |
| Period of maximum EPDS | 280 | 1.01(0.99 – 1.04) | 0.286 | 267 | 1.01(0.99 – 1.03) | 0.247 | 260 | 1.01(0.99 – 1.03) | 0.222 |
| Anxiety |  |  |  |  |  |  |  |  |  |
| STAI state | 281 | 1.02(0.99 – 1.04) | 0.220 | 268 | 1.00(0.98 – 1.03) | 0.714 | 261 | 1.00(0.98 – 1.02) | 0.841 |
| Period of maximum STAI state | 281 | 1.01(0.99 – 1.04) | 0.386 | 268 | 1.00(0.98 – 1.02) | 0.910 | 261 | 1.00(0.98 – 1.01) | 0.655 |
| STAI trait | 281 | 1.01(0.98 – 1.04) | 0.686 | 268 | 1.00(0.97 – 1.03) | 0.933 | 261 | 0.99(0.97 – 1.02) | 0.648 |
| STAI trait >=40 | 281 | 1.3(0.7 – 2.4) | 0.340 | 268 | 1.3(0.8 – 2.1) | 0.303 | 261 | 1.2(0.8 – 1.9) | 0.359 |
| Period of maximum STAI trait | 281 | 1.00(0.97 – 1.03) | 0.990 | 268 | 0.99(0.97 – 1.01) | 0.419 | 261 | 0.99(0.98 – 1.01) | 0.490 |
| PAQ | 260 | 1.0(0.6 – 1.6) | 0.998 | 249 | 0.89(0.60 – 1.33) | 0.579 | 242 | 0.86(0.60 – 1.25) | 0.435 |
| Period of maximum PAQ | 260 | 1.00(0.97 – 1.03) | 0.983 | 249 | 0.99(0.97 – 1.01) | 0.361 | 242 | 0.99(0.97 – 1.01) | 0.568 |
| Social support |  |  |  |  |  |  |  |  |  |
| MSPSS emotional support from partner | 261 | 0.97(0.59 – 1.62) | 0.920 | 250 | 0.94(0.62 – 1.41) | 0.752 | 243 | 1.1(0.7 – 1.6) | 0.688 |
| Period of maximum MSPSS emotional support from partner | 261 | 1.00(0.97 – 1.04) | 0.864 | 250 | 1.00(0.98 – 1.03) | 0.729 | 243 | 1.01(0.98 – 1.03) | 0.564 |
| MSPSS emotional support from family | 261 | 0.96(0.65 – 1.41) | 0.819 | 250 | 0.87(0.64 – 1.18) | 0.364 | 243 | 1.0(0.8 – 1.4) | 0.915 |
| Period of maximum MSPSS emotional support from family | 261 | 1.00(0.97 – 1.03) | 0.776 | 250 | 1.01(0.98 – 1.04) | 0.529 | 243 | 1.01(0.99 – 1.04) | 0.326 |
| MSPSS emotional support from friend | 261 | 0.98(0.69 – 1.41) | 0.930 | 250 | 1.0(0.8 – 1.4) | 0.887 | 243 | 0.98(0.75 – 1.29) | 0.891 |
| Period of maximum MSPSS emotional support from friend | 261 | 0.99(0.96 – 1.01) | 0.338 | 250 | 1.00(0.98 – 1.03) | 0.879 | 243 | 1.01(0.98 – 1.03) | 0.566 |
| MSPSS practical support from family | 261 | 1.1(0.7 – 1.6) | 0.771 | 250 | 0.89(0.65 – 1.22) | 0.466 | 243 | 1.0(0.7 – 1.4) | 0.969 |
| Period of maximum MSPSS practical support from family | 261 | 0.99(0.96 – 1.02) | 0.473 | 250 | 1.00(0.98 – 1.03) | 0.835 | 243 | 1.00(0.98 – 1.03) | 0.782 |
| MSPSS practical support from friend | 261 | 0.95(0.67 – 1.35) | 0.773 | 250 | 1.0(0.8 – 1.4) | 0.940 | 243 | 1.0(0.8 – 1.4) | 0.759 |
| Period of maximum MSPSS practical support from friend | 261 | 0.98(0.96 – 1.01) | 0.210 | 250 | 1.00(0.98 – 1.02) | 0.988 | 243 | 1.00(0.98 – 1.03) | 0.707 |
| **Post natal** |  |  |  |  |  |  |  |  |  |
| General stress |  |  |  |  |  |  |  |  |  |
| PSS | NA | NA | NA | 156 | 0.99(0.94 – 1.05) | 0.828 | 151 | 0.99(0.94 – 1.04) | 0.608 |
| PSS  0-13  14-26  >=27 | NA | NA | NA | 156 | 1.0  1.2(0.6 – 2.3)  0.85(0.11 – 6.47) | 0.607  0.872 | 151 | 1.0  0.89(0.50 – 1.58)  0.68(0.09 – 5.11) | 0.698  0.711 |
| Depression |  |  |  |  |  |  |  |  |  |
| BDI | 200^a^ | 0.99(0.94 – 1.03)^a^ | 0.568^a^ | 224 | 0.99(0.96 – 1.03) | 0.770 | 218 | 0.99(0.96 – 1.03) | 0.729 |
| BDI  0-13  14-19  >=20 | 200^a^ | 1.0  0.57(0.14 – 2.41) ^a^  0.46(0.11 – 1.93) ^a^ | 0.449 ^a^  0.288 ^a^ | 224 | 1.0  0.72(0.28 – 1.82)  0.91(0.41 – 2.01) | 0.485  0.809 | 218 | 1.0  0.95(0.45 – 2.00)  0.80(0.36 – 1.76) | 0.886  0.578 |
| Period of maximum BDI | NA | NA | NA | 224 | 1.1(1.0 – 1.4) | 0.142 | 218 | 1.2(1.0 – 1.4) | 0.069 |
| EPDS | 200^a^ | 1.05(0.98 – 1.12)^a^ | 0.167^a^ | 224 | 1.04(0.98 – 1.09) | 0.191 | 218 | 1.02(0.97 – 1.07) | 0.469 |
| EPDS>=13 | 200^a^ | 0.62(0.15 – 2.60)^a^ | 0.514^a^ | 224 | 1.1(0.5 – 2.3) | 0.802 | 218 | 1.0(0.5 – 2.1) | 0.936 |
| Period of maximum EPDS | NA | NA | NA | 224 | 1.1(0.9 – 1.3) | 0.281 | 218 | 1.1(1.0 – 1.3) | 0.163 |
| Anxiety |  |  |  |  |  |  |  |  |  |
| STAI state | 200^a^ | 1.02(0.99 – 1.05)^a^ | 0.257^a^ | 223 | 1.01(0.98 – 1.03) | 0.518 | 217 | 1.00(0.98 – 1.02) | 0.820 |
| Period of maximum STAI state | NA | NA | NA | 223 | 1.0(0.8 – 1.2) | 0.925 | 217 | 1.1(0.9 – 1.3) | 0.229 |
| STAI trait | 200^a^ | 1.01(0.98 – 1.05)^a^ | 0.353^a^ | 223 | 1.01(0.98 – 1.03) | 0.538 | 217 | 1.0(0.98 – 1.03) | 0.864 |
| STAI trait >=40 | 200^a^ | 1.6(0.9 – 3.2)^a^ | 0.193^a^ | 223 | 1.3(0.8 – 2.2) | 0.300 | 217 | 1.1(0.7 – 1.8) | 0.655 |
| Period of maximum STAI trait | NA | NA | NA | 223 | 0.91(0.77 – 1.09) | 0.328 | 217 | 1.0(0.9 – 1.2) | 0.995 |
| Social support |  |  |  |  |  |  |  |  |  |
| MSPSS emotional support from partner | NA | NA | NA | 165 | 0.78(0.57 – 1.09) | 0.142 | 160 | 0.87(0.64 – 1.18) | 0.369 |
| MSPSS emotional support from family | NA | NA | NA | 165 | 0.92(0.67 – 1.25) | 0.590 | 160 | 1.0(0.7 – 1.3) | 0.996 |
| MSPSS emotional support from friend | NA | NA | NA | 164 | 1.1(0.8 – 1.6) | 0.525 | 159 | 1.1(0.8 – 1.5) | 0.493 |
| MSPSS practical support from family | NA | NA | NA | 165 | 0.91(0.65 – 1.28) | 0.604 | 160 | 0.95(0.70 – 1.29) | 0.731 |
| MSPSS practical support from friend | NA | NA | NA | 165 | 1.0(0.8 – 1.4) | 0.778 | 160 | 1.0(0.8 – 1.3) | 0.955 |
| Life event |  |  |  |  |  |  |  |  |  |
| LES positive | NA | NA | NA | 159 | 1.0(0.9 – 1.1) | 0.918 | 156 | 1.00(0.95 – 1.05) | 0.987 |
| LES negative | NA | NA | NA | 159 | 0.98(0.93 – 1.04) | 0.560 | 156 | 0.97(0.92 – 1.03) | 0.352 |

BDI-II: Beck Depression Inventory-II; EPDS: Edinburgh Postnatal Depression Scale; GHQ: General Health Questionnaire; LES: Life Experiences Survey; MSPSS: Multidimensional Scale of Perceived Social Support; PAQ: Pregnancy Anxiety Questionnaire; PES: General Health Questionnaire; PSS: Perceived Stress Scale; STAI: State-Trait Anxiety Inventory

CI: confidence interval; RR: risk ratio

RR = 1.0 is the reference category.

Significant p value in bold

NA: not applicable (period is constant/stress accessed at the same time point with the outcome)

^a^Month 3 post natal stress

**Supplementary Table 2.** Univariate poisson regression of rhinitis by 6, 12 and 18 months.

|  | 6 months | | | 12 months | | | 18 months | | |
| --- | --- | --- | --- | --- | --- | --- | --- | --- | --- |
|  | n | RR (95% CI) | p-value | n | RR (95% CI) | p-value | n | RR (95% CI) | p-value |
| Ethnicity  Chinese  Malay  Indian  Mix | 307 | 1.0  1.4(0.8 – 2.3)  1.1(0.5 – 2.4)  1.9(0.9 – 4.0) | 0.247  0.901  0.074 | 296 | 1.0  1.4(0.9 – 2.2)  1.1(0.6 – 2.3)  1.6(0.8 – 3.1) | 0.092  0.701  0.188 | 288 | 1.0  1.3(0.8 – 1.9)  1.1(0.6 – 2.2)  1.3(0.7 – 2.6) | 0.273  0.712  0.411 |
| Education ≥ 12 years | 307 | 0.78(0.41 – 1.49) | 0.449 | 296 | 0.96(0.52 – 1.77) | 0.887 | 288 | 0.93(0.53 – 1.64) | 0.799 |
| Maternal age | 307 | 0.97(0.91 – 1.03) | 0.260 | 296 | 0.99(0.94 – 1.04) | 0.639 | 288 | 1.0(0.95 – 1.04) | 0.885 |
| Maternal allergy | 307 | 1.2(0.8 – 1.8) | 0.468 | 296 | 1.2(0.8 – 1.7) | 0.468 | 288 | 1.2(0.8 – 1.8) | 0.280 |
| Parous | 306 | 1.4(1.0 – 2.0) | 0.084 | 295 | 1.5(1.0 – 2.0) | **0.025** | 287 | 1.3(0.9 – 1.7) | 0.151 |
| Smoking during pregnancy | 290 | 1.5(0.2 – 10.4) | 0.709 | 278 | 1.0(0.1 – 7.4) | 0.977 | 270 | 0.89(0.12 – 6.34) | 0.905 |
| Male infant | 307 | 1.1(0.8 – 1.6) | 0.563 | 295 | 1.0(0.7 – 1.4) | 0.853 | 287 | 1.0(0.7 – 1.4) | 0.998 |
| Gestational age at birth | 305 | 0.94(0.83 – 1.06) | 0.302 | 293 | 0.95(0.85 – 1.05) | 0.322 | 285 | 0.96(0.87 – 1.07) | 0.505 |
| **Preconception** |  |  |  |  |  |  |  |  |  |
| General stress |  |  |  |  |  |  |  |  |  |
| GHQ | 174 | 1.03(0.98 – 1.08) | 0.310 | 167 | 1.04(0.99 – 1.08) | 0.109 | 160 | 1.03(0.99 – 1.08) | 0.121 |
| PSS | 172 | 1.03(0.98 – 1.07) | 0.265 | 165 | 1.03(0.99 – 1.08) | 0.104 | 158 | 1.03(0.99 – 1.07) | 0.118 |
| PSS  0-13  14-26  >=27 | 172 | 1.0  1.0(0.6 – 1.7)  0.97(0.13 – 7.20) | 0.986  0.975 | 165 | 1.0  1.2(0.7 – 2.0)  0.86(0.12 – 6.41) | 0.459  0.886 | 158 | 1.0  1.2(0.7 – 1.9)  1.4(0.3 – 6.0) | 0.500  0.622 |
| Depression |  |  |  |  |  |  |  |  |  |
| BDI | 168 | 1.01(0.98 – 1.04) | 0.522 | 158 | 1.01(0.99 – 1.04) | 0.304 | 149 | 1.02(0.99 – 1.05) | 0.120 |
| BDI  0-13  14-19  >=20 | 168 | 1.0  1.3(0.5 – 3.4)  1.3(0.6 – 2.7) | 0.547  0.468 | 158 | 1.0  1.1(0.4 – 2.7)  1.4(0.8 – 2.6) | 0.882  0.284 | 149 | 1.0  1.4(0.6 – 3.0)  1.5(0.9 – 2.7) | 0.450  0.151 |
| Period of maximum BDI | 168 | 1.0(0.9 – 1.2) | 0.803 | 158 | 0.98(0.84 – 1.14) | 0.780 | 149 | 0.97(0.83 – 1.12) | 0.667 |
| EPDS | 205 | 1.04(0.98 – 1.10) | 0.162 | 195 | 1.04(0.99 – 1.09) | 0.161 | 186 | 1.03(0.98 – 1.08) | 0.238 |
| EPDS>=13 | 205 | 1.4(0.7 – 2.6) | 0.343 | 195 | 1.6(0.9 – 2.7) | 0.118 | 186 | 1.5(0.9 – 2.5) | 0.137 |
| Period of maximum EPDS | 205 | 1.1(0.9 – 1.2) | 0.299 | 195 | 1.0(0.9 – 1.2) | 0.553 | 186 | 1.1(0.9 – 1.2) | 0.485 |
| Anxiety |  |  |  |  |  |  |  |  |  |
| STAI state | 204 | 1.00(0.98 – 1.03) | 0.727 | 194 | 1.00(0.98 – 1.03) | 0.673 | 185 | 1.00(0.98 – 1.02) | 0.893 |
| Period of maximum STAI state | 204 | 1.0(0.9 – 1.1) | 0.939 | 194 | 1.0(0.9 – 1.1) | 0.808 | 185 | 1.0(0.9 – 1.1) | 0.961 |
| STAI trait | 205 | 1.01(0.98 – 1.04) | 0.483 | 195 | 1.01(0.99 – 1.04) | 0.256 | 186 | 1.01(0.99 – 1.04) | 0.318 |
| STAI trait >=40 | 205 | 1.5(0.9 – 2.4) | 0.088 | 195 | 1.5(1.0 – 2.2) | 0.069 | 186 | 1.3(0.9 – 1.9) | 0.239 |
| Period of maximum STAI trait | 205 | 1.0(0.9 – 1.1) | 0.975 | 195 | 1.0(0.9 – 1.1) | 0.808 | 186 | 1.0(0.9 – 1.1) | 0.880 |
| Social support |  |  |  |  |  |  |  |  |  |
| MSPSS emotional support from partner | 173 | 1.0(0.7 – 1.4) | 0.938 | 166 | 0.96(0.74 – 1.24) | 0.744 | 159 | 0.99(0.77 – 1.27) | 0.925 |
| MSPSS emotional support from family | 173 | 0.93(0.73 – 1.19) | 0.570 | 166 | 0.93(0.75 – 1.16) | 0.542 | 159 | 0.94(0.76 – 1.16) | 0.546 |
| MSPSS emotional support from friend | 173 | 0.99(0.76 – 1.29) | 0.955 | 166 | 0.93(0.74 – 1.17) | 0.527 | 159 | 0.98(0.79 – 1.22) | 0.858 |
| MSPSS practical support from family | 173 | 0.95(0.75 – 1.22) | 0.705 | 166 | 1.0(0.8 – 1.2) | 0.982 | 159 | 1.00(0.81 – 1.22) | 0.968 |
| MSPSS practical support from friend | 173 | 0.96(0.74 – 1.25) | 0.776 | 166 | 0.88(0.70 – 1.10) | 0.261 | 159 | 0.93(0.75 – 1.15) | 0.497 |
| Life event |  |  |  |  |  |  |  |  |  |
| LES positive | 164 | 1.02(0.98 – 1.06) | 0.359 | 157 | 1.00(0.96 – 1.04) | 0.922 | 150 | 1.00(0.96 – 1.04) | 0.875 |
| LES negative | 164 | 1.01(0.96 – 1.06) | 0.786 | 157 | 1.02(0.98 – 1.07) | 0.311 | 150 | 1.02(0.98 – 1.07) | 0.290 |
| **Pregnancy** |  |  |  |  |  |  |  |  |  |
| General stress |  |  |  |  |  |  |  |  |  |
| PES Hassles/Uplifts frequency ratio | 272 | 0.86(0.60 – 1.23) | 0.399 | 262 | 0.90(0.66 – 1.22) | 0.487 | 255 | 0.91(0.69 – 1.21) | 0.510 |
| Period of maximum PES Hassles/Uplifts frequency ratio | 272 | 0.99(0.97 – 1.01) | 0.270 | 262 | 0.99(0.98 – 1.01) | 0.298 | 255 | 1.00(0.98 – 1.01) | 0.693 |
| PES Hassles/Uplifts intensity ratio | 272 | 1.4(0.8 – 2.5) | 0.296 | 262 | 1.3(0.8 – 2.2) | 0.298 | 255 | 1.1(0.7 – 1.9) | 0.675 |
| Period of maximum PES Hassles/Uplifts intensity ratio | 272 | 0.98(0.97 – 1.00) | 0.092 | 262 | 0.99(0.98 – 1.01) | 0.274 | 255 | 1.00(0.98 – 1.01) | 0.589 |
| PSS | 271 | 1.01(0.98 – 1.05) | 0.467 | 261 | 1.03(1.00 – 1.06) | 0.060 | 254 | 1.02(0.99 – 1.05) | 0.200 |
| PSS  0-13  14-26  >=27 | 271 | 1.0  0.97(0.6 – 1.56)  1.2(0.4 – 3.1) | 0.888  0.771 | 261 | 1.0  1.1(0.7 – 1.7)  1.6(0.7 – 3.7) | 0.766  0.274 | 254 | 1.0  1.1(0.7 – 1.6)  1.4(0.6 – 3.1) | 0.715  0.451 |
| Period of maximum PSS | 271 | 1.00(0.98 – 1.01) | 0.700 | 261 | 1.00(0.99 – 1.02) | 0.729 |  | 1.00(0.99 – 1.02) | 0.621 |
| Depression |  |  |  |  |  |  |  |  |  |
| BDI | 287 | 1.02(0.99 – 1.06) | 0.218 | 275 | 1.02(1.00 – 1.04) | **0.039** | 267 | 1.02(1.00 – 1.04) | 0.114 |
| BDI  0-13  14-19  >=20 | 287 | 1.0  1.3(0.8 – 2.1)  1.3(0.8 – 2.2) | 0.340  0.347 | 275 | 1.0  1.4(0.9 – 2.1)  1.5(0.9 – 2.3) | 0.152  0.099 | 267 | 1.0  1.2(0.8 – 1.9)  1.3(0.9 – 2.0) | 0.323  0.204 |
| Period of maximum BDI | 287 | 1.00(0.98 – 1.02) | 0.850 | 275 | 1.00(0.98 – 1.02) | 0.999 | 267 | 1.00(0.99 – 1.02) | 0.708 |
| EPDS | 288 | 1.03(0.99 – 1.07) | 0.163 | 276 | 1.04(1.00 – 1.07) | **0.044** | 268 | 1.02(0.99 – 1.05) | 0.222 |
| EPDS>=13 | 288 | 1.3(0.8 – 2.1) | 0.303 | 276 | 1.4(1.0 – 2.1) | 0.075 | 268 | 1.3(0.9 – 1.9) | 0.236 |
| Period of maximum EPDS | 288 | 0.99(0.98 – 1.01) | 0.536 | 276 | 1.00(0.98 – 1.01) | 0.781 | 268 | 1.00(0.99 – 1.01) | 0.998 |
| Anxiety |  |  |  |  |  |  |  |  |  |
| STAI state | 289 | 1.01(0.99 – 1.02) | 0.524 | 277 | 1.01(0.99 – 1.03) | 0.228 | 269 | 1.00(0.99 – 1.02) | 0.654 |
| Period of maximum STAI state | 289 | 0.99(0.98 – 1.01) | 0.557 | 277 | 0.99(0.98 – 1.01) | 0.495 | 269 | 1.00(0.98 – 1.01) | 0.740 |
| STAI trait | 289 | 1.01(0.99 – 1.03) | 0.416 | 277 | 1.01(1.00 – 1.03) | 0.144 | 269 | 1.01(0.99 – 1.03) | 0.355 |
| STAI trait >=40 | 289 | 1.2(0.8 – 1.8) | 0.398 | 277 | 1.2(0.9 – 1.7) | 0.240 | 269 | 1.1(0.8 – 1.5) | 0.584 |
| Period of maximum STAI trait | 289 | 1.00(0.98 – 1.02) | 0.935 | 277 | 1.00(0.99 – 1.02) | 0.664 | 269 | 1.00(0.99 – 1.01) | 0.957 |
| PAQ | 268 | 1.4(1.0 – 2.0) | **0.024** | 258 | 1.4(1.1 – 1.9) | **0.015** | 251 | 1.3(1.0 – 1.8) | **0.028** |
| Period of maximum PAQ | 268 | 1.00(0.98 – 1.02) | 0.923 | 258 | 1.01(0.99 – 1.02) | 0.375 | 251 | 1.01(1.00 – 1.03) | 0.137 |
| Social support |  |  |  |  |  |  |  |  |  |
| MSPSS emotional support from partner | 269 | 0.92(0.66 – 1.28) | 0.621 | 259 | 0.88(0.67 – 1.17) | 0.381 | 252 | 0.93(0.71 – 1.22) | 0.613 |
| Period of maximum MSPSS emotional support from partner | 269 | 0.98(0.96 – 1.00) | **0.016** | 259 | 0.98(0.96 – 1.00) | **0.021** | 252 | 0.98(0.97 – 1.00) | **0.038** |
| MSPSS emotional support from family | 269 | 0.82(0.64 – 1.06) | 0.126 | 259 | 0.83(0.67 – 1.03) | 0.097 | 252 | 0.88(0.72 – 1.09) | 0.254 |
| Period of maximum MSPSS emotional support from family | 269 | 0.99(0.97 – 1.00) | 0.144 | 259 | 0.99(0.97 – 1.01) | 0.320 | 252 | 0.99(0.97 – 1.00) | 0.170 |
| MSPSS emotional support from friend | 269 | 0.86(0.68 – 1.09) | 0.212 | 259 | 0.88(0.71 – 1.08) | 0.207 | 252 | 0.93(0.76 – 1.13) | 0.476 |
| Period of maximum MSPSS emotional support from friend | 269 | 0.98(0.97 – 1.00) | 0.089 | 259 | 0.99(0.98 – 1.01) | 0.361 | 252 | 0.99(0.98 – 1.01) | 0.417 |
| MSPSS practical support from family | 269 | 0.86(0.67 – 1.11) | 0.260 | 259 | 0.84(0.67 – 1.05) | 0.121 | 252 | 0.84(0.68 – 1.05) | 0.120 |
| Period of maximum MSPSS practical support from family | 269 | 0.99(0.97 – 1.00) | 0.142 | 259 | 0.99(0.98 – 1.01) | 0.294 | 252 | 0.99(0.97 – 1.00) | 0.171 |
| MSPSS practical support from friend | 269 | 0.82(0.65 – 1.03) | 0.082 | 259 | 0.86(0.71 – 1.06) | 0.158 | 252 | 0.93(0.77 – 1.13) | 0.466 |
| Period of maximum MSPSS practical support from friend | 269 | 1.00(0.98 – 1.02) | 0.709 | 259 | 1.00(0.98 – 1.01) | 0.611 | 252 | 1.00(0.98 – 1.01) | 0.709 |
| **Post natal** |  |  |  |  |  |  |  |  |  |
| General stress |  |  |  |  |  |  |  |  |  |
| PSS | NA | NA | NA | 163 | 1.01(0.97 – 1.05) | 0.664 | 158 | 1.01(0.97 – 1.05) | 0.717 |
| PSS  0-13  14-26  >=27 | NA | NA | NA | 163 | 1.0  0.98(0.59 – 1.63)  1.5(0.4 – 4.9) | 0.934  0.537 | 158 | 1.0  0.99(0.61 – 1.62)  1.4(0.5 – 4.1) | 0.974  0.498 |
| Depression |  |  |  |  |  |  |  |  |  |
| BDI | 206^a^ | 1.02(0.99 – 1.04)^a^ | 0.161^a^ | 234 | 1.02(0.99 – 1.04) | 0.174 | 226 | 1.02(0.99 – 1.04) | 0.146 |
| BDI  0-13  14-19  >=20 | 206^a^ | 1.0  1.8(0.9 – 3.5)^a^  1.3(0.7 – 2.7)^a^ | 0.103^a^  0.425^a^ | 234 | 1.0  1.3(0.8 – 2.3)  1.2(0.7 – 2.1) | 0.274  0.466 | 226 | 1.0  1.3(0.8 – 2.1)  1.3(0.8 – 2.1) | 0.332  0.346 |
| Period of maximum BDI | NA | NA | NA | 234 | 0.94(0.83 – 1.07) | 0.338 | 226 | 0.95(0.84 – 1.07) | 0.373 |
| EPDS | 206^a^ | 1.01(0.97 – 1.07)^a^ | 0.558^a^ | 234 | 1.00(0.96 – 1.04) | 0.893 | 226 | 1.00(0.97 – 1.04) | 0.820 |
| EPDS>=13 | 206^a^ | 1.0(0.4 – 2.3)^a^ | 0.993^a^ | 234 | 0.96(0.55 – 1.69) | 0.889 | 226 | 1.0(0.6 – 1.7) | 0.939 |
| Period of maximum EPDS | NA | NA | NA | 234 | 0.95(0.84 – 1.08) | 0.438 | 226 | 0.96(0.85 – 1.09) | 0.527 |
| Anxiety |  |  |  |  |  |  |  |  |  |
| STAI state | 206^a^ | 1.02(1.00 – 1.05)^a^ | 0.170^a^ | 233 | 1.01(0.99 – 1.02) | 0.389 | 225 | 1.00(0.99 – 1.02) | 0.601 |
| Period of maximum STAI state | NA | NA | NA | 233 | 0.94(0.83 – 1.07) | 0.363 | 225 | 0.94(0.83 – 1.07) | 0.342 |
| STAI trait | 206^a^ | 1.02(0.99 – 1.04)^a^ | 0.149^a^ | 233 | 1.01(0.99 – 1.03) | 0.321 | 225 | 1.01(0.99 – 1.02) | 0.544 |
| STAI trait >=40 | 206^a^ | 1.6(1.0 – 2.7)^a^ | 0.052^a^ | 233 | 1.3(0.9 – 2.0) | 0.138 | 225 | 1.2(0.8 – 1.7) | 0.309 |
| Period of maximum STAI trait | NA | NA | NA | 233 | 0.92(0.8 – 1.04) | 0.178 | 225 | 0.93(0.82 – 1.05) | 0.258 |
| Social support |  |  |  |  |  |  |  |  |  |
| MSPSS emotional support from partner | NA | NA | NA | 170 | 0.85(0.65 – 1.10) | 0.214 | 165 | 0.88(0.68 – 1.14) | 0.343 |
| MSPSS emotional support from family | NA | NA | NA | 170 | 0.90(0.71 – 1.14) | 0.365 | 165 | 0.91(0.73 – 1.15) | 0.441 |
| MSPSS emotional support from friend | NA | NA | NA | 169 | 0.91(0.72 – 1.15) | 0.431 | 164 | 0.94(0.75 – 1.17) | 0.568 |
| MSPSS practical support from family | NA | NA | NA | 170 | 0.91(0.70 – 1.17) | 0.460 | 165 | 0.94(0.73 – 1.21) | 0.636 |
| MSPSS practical support from friend | NA | NA | NA | 170 | 0.86(0.68 – 1.09) | 0.218 | 165 | 0.91(0.72 – 1.14) | 0.394 |
| Life event |  |  |  |  |  |  |  |  |  |
| LES positive | NA | NA | NA | 164 | 1.01(0.97 – 1.05) | 0.558 | 160 | 1.01(0.97 – 1.05) | 0.670 |
| LES negative | NA | NA | NA | 164 | 1.02(0.98 – 1.06) | 0.352 | 160 | 1.01(0.97 – 1.05) | 0.559 |

BDI-II: Beck Depression Inventory-II; EPDS: Edinburgh Postnatal Depression Scale; GHQ: General Health Questionnaire; LES: Life Experiences Survey; MSPSS: Multidimensional Scale of Perceived Social Support; PAQ: Pregnancy Anxiety Questionnaire; PES: General Health Questionnaire; PSS: Perceived Stress Scale; STAI: State-Trait Anxiety Inventory

CI: confidence interval; RR: risk ratio

RR = 1.0 is the reference category.

Significant p value in bold

NA: not applicable (period is constant/stress accessed at the same time point with the outcome)

^a^Month 3 post natal stress

**Supplementary Table 3.** Univariate poisson regression of wheeze by 6, 12 and 18 months.

|  | 6 months | | | 12 months | | | 18 months | | |
| --- | --- | --- | --- | --- | --- | --- | --- | --- | --- |
|  | n | RR (95% CI) | p-value | n | RR (95% CI) | p-value | n | RR (95% CI) | p-value |
| Ethnicity  Chinese  Malay  Indian  Mix | 299 | 1.0  1.8(0.4 – 9)  2.4(0.3 – 19.7)  2.9(0.4 – 24.3) | 0.468  0.423  0.321 | 277 | 1.7(0.2 – 12.4)  2.5(0.3 – 20.4)  1.0  1.5(0.1 – 23.3) | 0.617  0.404  0.791 | 258 | 1.8(0.2 – 13)  2.3(0.3 – 19.4)  1.0  1.4(0.1 – 22.4) | 0.576  0.433  0.812 |
| Education ≥ 12 years | 299 | 0.33(0.07 – 1.57) | 0.165 | 277 | 0.43(0.17 – 1.13) | 0.086 | 258 | 0.47(0.18 – 1.22) | 0.121 |
| Maternal age | 299 | 0.89(0.73 – 1.08) | 0.245 | 277 | 0.95(0.86 – 1.07) | 0.410 | 258 | 0.94(0.84 – 1.05) | 0.258 |
| Maternal allergy | 299 | 1.6(0.3 – 7.5) | 0.558 | 277 | 1.2(0.5 – 2.8) | 0.645 | 258 | 1.4(0.6 – 3.2) | 0.446 |
| Parous | 298 | 1.5(0.4 – 5.2) | 0.519 | 276 | 1.4(0.7 – 2.9) | 0.336 | 257 | 1.3(0.7 – 2.6) | 0.426 |
| Smoking during pregnancy | 283 | NA | NA | 261 | 4.8(0.7 – 35.3) | 0.124 | 244 | 8.1(1.1 – 59.4) | **0.040** |
| Male infant | 299 | 0.20(0.04 – 0.96) | **0.044** | 277 | 1.3(0.6 – 2.6) | 0.547 | 258 | 1.5(0.7 – 3.0) | 0.303 |
| Gestational age at birth | 297 | 0.78(0.61 – 0.98) | **0.036** | 275 | 1.0(0.8 – 1.3) | 0.978 | 256 | 0.98(0.78 – 1.25) | 0.891 |
| **Preconception** |  |  |  |  |  |  |  |  |  |
| General stress |  |  |  |  |  |  |  |  |  |
| GHQ | 172 | 1.1(1.0– 1.3) | 0.065 | 158 | 1.13(1.06 – 1.21) | **<0.001** | 146 | 1.12(1.05 – 1.20) | **<0.001** |
| PSS | 170 | 1.0(0.9 – 1.2) | 0.559 | 156 | 1.1(1.0 – 1.2) | **0.045** | 145 | 1.1(1.0 – 1.2) | **0.047** |
| PSS >=14 | 170 | 1.4(0.3 – 7.0) | 0.711 | 156 | 1.4(0.5 – 4.0) | 0.483 | 145 | 1.3(0.5 – 3.4) | 0.570 |
| Depression |  |  |  |  |  |  |  |  |  |
| BDI | 166 | 1.04(0.95 – 1.13) | 0.396 | 150 | 1.07(1.02 – 1.12) | **0.005** | 136 | 1.06(1.01 – 1.10) | **0.018** |
| BDI  0-13  14-19  >=20 | 166 | 1.0  2.7(0.3 – 24.6)  3.0(0.5 – 16.4) | 0.366  0.205 | 150 | 1.0  1.1(0.1 – 8.3)  3.7(1.4 – 9.8) | 0.953  **0.008** | 136 | 1.0  0.82(0.11 – 6.23) 2.9(1.1 – 7.2) | 0.844  **0.025** |
| Period of maximum BDI | 166 | 1.1(0.7 – 1.6) | 0.689 | 150 | 1.0(0.8 – 1.3) | 0.945 | 136 | 0.98(0.75 – 1.29) | 0.897 |
| EPDS | 202 | 1.1(0.9 – 1.2) | 0.568 | 183 | 1.1(1.0 – 1.2) | **0.011** | 169 | 1.1(1.0 – 1.2) | **0.030** |
| EPDS>=13 | 202 | 1.2(0.1 – 9.8) | 0.878 | 183 | 1.9(0.6 – 5.8) | 0.240 | 169 | 1.6(0.5 – 4.6) | 0.413 |
| Period of maximum EPDS | 202 | 1.2(0.8 – 1.7) | 0.395 | 183 | 1.1(0.8 – 1.4) | 0.484 | 169 | 1.1(0.8 – 1.4) | 0.527 |
| Anxiety |  |  |  |  |  |  |  |  |  |
| STAI state | 201 | 1.0(0.9 – 1.1) | 0.925 | 182 | 1.05(1.0 – 1.1) | **0.030** | 168 | 1.04(1.00 – 1.09) | **0.042** |
| Period of maximum STAI state | 201 | NA | NA | 182 | 1.1(1.0 – 1.3) | 0.163 | 168 | 1.1(0.9 – 1.3) | 0.237 |
| STAI trait | 202 | 1.0(0.9 – 1.1) | 0.904 | 183 | 1.06(1.01 – 1.11) | **0.019** | 169 | 1.06(1.01 – 1.11) | **0.013** |
| STAI trait >=40 | 202 | 1.0(0.2 – 4.6) | 0.967 | 183 | 2.0(0.8 – 4.9) | 0.124 | 169 | 2.0(0.9 – 4.7) | 0.094 |
| Period of maximum STAI trait | 202 | 0.91(0.55 – 1.50) | 0.706 | 183 | 1.1(0.9 – 1.3) | 0.292 | 169 | 1.1(0.9 – 1.3) | 0.339 |
| Social support |  |  |  |  |  |  |  |  |  |
| MSPSS emotional support from partner | 171 | 0.93(0.38 – 2.28) | 0.883 | 157 | 1.0(0.6 – 1.8) | 0.939 | 146 | 0.84(0.55 – 1.31) | 0.448 |
| MSPSS emotional support from family | 171 | 1.3(0.5 – 3.1) | 0.603 | 157 | 0.69(0.47 – 1.01) | 0.057 | 146 | 0.64(0.45 – 0.92) | **0.015** |
| MSPSS emotional support from friend | 171 | 0.75(0.36 – 1.56) | 0.444 | 157 | 0.79(0.52 – 1.20) | 0.263 | 146 | 0.73(0.49 – 1.09) | 0.123 |
| MSPSS practical support from family | 171 | 1.1(0.5 – 2.4) | 0.845 | 157 | 0.69(0.47 – 1.01) | 0.059 | 146 | 0.66(0.46 – 0.94) | **0.023** |
| MSPSS practical support from friend | 171 | 0.97(0.43 – 2.19) | 0.941 | 157 | 0.86(0.55 – 1.34) | 0.500 | 146 | 0.82(0.54 – 1.26) | 0.368 |
| Life event |  |  |  |  |  |  |  |  |  |
| LES positive | 162 | 1.0(0.9 – 1.1) | 0.770 | 147 | 0.97(0.89 – 1.06) | 0.471 | 136 | 0.95(0.87 – 1.04) | 0.251 |
| LES negative | 162 | 1.1(1.0 – 1.2) | 0.102 | 147 | 1.06(0.99 – 1.15) | 0.098 | 136 | 1.04(0.97 – 1.12) | 0.270 |
| **Pregnancy** |  |  |  |  |  |  |  |  |  |
| General stress |  |  |  |  |  |  |  |  |  |
| PES Hassles/Uplifts frequency ratio | 267 | 0.78(0.23 – 2.62) | 0.683 | 250 | 1.2(0.7 – 1.9) | 0.524 | 234 | 1.1(0.7 – 1.7) | 0.735 |
| Period of maximum PES Hassles/Uplifts frequency ratio | 267 | 0.97(0.92 – 1.03) | 0.329 | 250 | 1.00(0.97 – 1.03) | 0.842 | 234 | 1.01(0.98 – 1.04) | 0.574 |
| PES Hassles/Uplifts intensity ratio | 267 | 1.9(0.4 – 9.8) | 0.463 | 250 | 1.3(0.4 – 3.9) | 0.652 | 234 | 1.2(0.4 – 3.5) | 0.700 |
| Period of maximum PES Hassles/Uplifts intensity ratio | 267 | 1.0(0.9 – 1.1) | 0.997 | 250 | 0.98(0.95 – 1.02) | 0.404 | 234 | 1.00(0.97 – 1.03) | 0.983 |
| PSS | 266 | 1.1(1.0 – 1.2) | **0.033** | 249 | 1.1(1.0 – 1.2) | **0.017** | 233 | 1.07(1.01 – 1.14) | **0.024** |
| PSS >=27 | 266 | 5.3(1.1 – 24.9) | **0.035** | 249 | 3.8(1.3 – 11.0) | **0.013** | 233 | 3.2(1.1 – 9.1) | **0.030** |
| Period of maximum PSS | 266 | 1.03(0.97 – 1.10) | 0.358 | 249 | 1.00(0.96 – 1.03) | 0.777 | 233 | 1.01(0.98 – 1.04) | 0.667 |
| Depression |  |  |  |  |  |  |  |  |  |
| BDI | 280 | 1.04(0.97 – 1.11) | 0.231 | 260 | 1.04(1.00 – 1.09) | **0.032** | 243 | 1.04(1.00 – 1.08) | 0.062 |
| BDI  0-13  14-19  >=20 | 280 | 1.0  1.5(0.3 – 7.8)  2.8(0.7 – 11.6) | 0.621  0.164 | 260 | 1.0  1.2(0.4 – 3.3)  2.5(1.1 – 5.7) | 0.695  **0.025** | 243 | 1.0  1.1(0.4 – 2.9)  2.6(1.2 – 5.7) | 0.900  **0.015** |
| Period of maximum BDI | 280 | 1.01(0.96 – 1.07) | 0.677 | 260 | 1.02(0.99 – 1.06) | 0.249 | 243 | 1.03(1.00 – 1.07) | 0.090 |
| EPDS | 281 | 1.1(1.0 – 1.2) | 0.104 | 261 | 1.08(1.01 – 1.15) | **0.025** | 244 | 1.07(1.00 – 1.14) | **0.038** |
| EPDS>=13 | 281 | 0.49(0.06 – 3.86) | 0.498 | 261 | 1.3(0.5 – 2.9) | 0.601 | 244 | 1.3(0.6 – 2.9) | 0.510 |
| Period of maximum EPDS | 281 | 1.02(0.97 – 1.08) | 0.441 | 261 | 1.01(0.98 – 1.05) | 0.450 | 244 | 1.02(0.99 – 1.06) | 0.147 |
| Anxiety |  |  |  |  |  |  |  |  |  |
| STAI state | 282 | 1.05(1.00 – 1.11) | **0.039** | 262 | 1.05(1.01 – 1.08) | **0.004** | 245 | 1.04(1.01 – 1.07) | **0.017** |
| Period of maximum STAI state | 282 | 0.99(0.94 – 1.04) | 0.624 | 262 | 1.00(0.97 – 1.03) | 0.978 | 245 | 1.01(0.98 – 1.04) | 0.613 |
| STAI trait | 282 | 1.05(0.99 – 1.11) | 0.106 | 262 | 1.04(1.00 – 1.08) | 0.054 | 245 | 1.03(1 – 1.07) | 0.084 |
| STAI trait >=40 | 282 | 4.2(0.9 – 19.7) | 0.071 | 262 | 2.4(1.1 – 5.3) | **0.028** | 245 | 2.0(1.0 – 4.2) | 0.058 |
| Period of maximum STAI trait | 282 | 1.01(0.96 – 1.07) | 0.684 | 262 | 1.00(0.97 – 1.03) | 0.963 | 245 | 1.01(0.98 – 1.04) | 0.522 |
| PAQ | 263 | 0.82(0.30 – 2.27) | 0.710 | 246 | 1.3(0.7 – 2.3) | 0.385 | 231 | 1.2(0.7 – 2.2) | 0.434 |
| Period of maximum PAQ | 263 | 1.01(0.95 – 1.07) | 0.798 | 246 | 1.03(1.00 – 1.07) | 0.077 | 231 | 1.04(1.00 – 1.07) | 0.050 |
| Social support |  |  |  |  |  |  |  |  |  |
| MSPSS emotional support from partner | 264 | 0.95(0.34 – 2.68) | 0.923 | 247 | 1.1(0.6 – 2.1) | 0.809 | 232 | 1.2(0.6 – 2.4) | 0.640 |
| Period of maximum MSPSS emotional support from partner | 264 | 0.99(0.93 – 1.05) | 0.723 | 247 | 1.03(0.98 – 1.08) | 0.271 | 232 | 1.03(0.98 – 1.08) | 0.208 |
| MSPSS emotional support from family | 264 | 1.2(0.5 – 2.9) | 0.718 | 247 | 0.93(0.58 – 1.49) | 0.770 | 232 | 0.90(0.57 – 1.41) | 0.637 |
| Period of maximum MSPSS emotional support from family | 264 | 1.03(0.95 – 1.11) | 0.483 | 247 | 1.02(0.98 – 1.06) | 0.354 | 232 | 1.03(0.98 – 1.07) | 0.222 |
| MSPSS emotional support from friend | 264 | 0.70(0.35 – 1.37) | 0.295 | 247 | 0.97(0.62 – 1.50) | 0.881 | 232 | 0.95(0.62 – 1.45) | 0.810 |
| Period of maximum MSPSS emotional support from friend | 264 | 0.97(0.92 – 1.02) | 0.189 | 247 | 1.03(0.99 – 1.07) | 0.193 | 232 | 1.02(0.99 – 1.06) | 0.239 |
| MSPSS practical support from family | 264 | 0.73(0.35 – 1.53) | 0.406 | 247 | 0.73(0.47 – 1.13) | 0.156 | 232 | 0.72(0.47 – 1.11) | 0.136 |
| Period of maximum MSPSS practical support from family | 264 | 1.01(0.95 – 1.07) | 0.799 | 247 | 1.03(0.99 – 1.07) | 0.210 | 232 | 1.03(0.99 – 1.08) | 0.101 |
| MSPSS practical support from friend | 264 | 0.71(0.36 – 1.40) | 0.328 | 247 | 0.99(0.64 – 1.52) | 0.953 | 232 | 1.0(0.7 – 1.6) | 0.938 |
| Period of maximum MSPSS practical support from friend | 264 | 1.01(0.95 – 1.08) | 0.659 | 247 | 1.03(0.99 – 1.07) | 0.148 | 232 | 1.02(0.99 – 1.06) | 0.217 |
| **Post natal** |  |  |  |  |  |  |  |  |  |
| General stress |  |  |  |  |  |  |  |  |  |
| PSS | NA | NA | NA | 154 | 1.04(0.96 – 1.14) | 0.328 | 143 | 1.03(0.96 – 1.12) | 0.415 |
| PSS  0-13  14-26  >=27 | NA | NA | NA | 154 | 1.0  0.56(0.18 – 1.73) 2.9(0.6 – 14.6) | 0.312  0.186 | 143 | 1.0  0.84(0.30 – 2.35) 2.7(0.5 – 13.5) | 0.733  0.220 |
| Depression |  |  |  |  |  |  |  |  |  |
| BDI | 201^a^ | 1.0(0.9 – 1.1)^a^ | 0.834^a^ | 219 | 1.03(0.99 – 1.07) | 0.174 | 203 | 1.02(0.98 – 1.07) | 0.265 |
| BDI >=14 | 201^a^ | 1.5(0.2 – 13.2)^a^ | 0.690^a^ | 219 | 1.7(0.6 – 4.6) | 0.278 | 203 | 1.5(0.6 – 3.9) | 0.428 |
| Period of maximum BDI | NA | NA | NA | 219 | 0.80(0.60 – 1.07) | 0.132 | 203 | 0.86(0.66 – 1.13) | 0.275 |
| EPDS | 201^a^ | 1.1(0.9 – 1.2)^a^ | 0.223^a^ | 219 | 1.0(0.9 – 1.1) | 0.799 | 203 | 1.0(0.9 – 1.1) | 0.916 |
| EPDS>=13 | 201^a^ | 2.0(0.2 – 17.4)^a^ | 0.517^a^ | 219 | 1.3(0.4 – 3.8) | 0.622 | 203 | 1.1(0.4 – 3.3) | 0.817 |
| Period of maximum EPDS | NA | NA | NA | 219 | 0.90(0.67 – 1.19) | 0.482 | 203 | 0.91(0.7 – 1.17) | 0.465 |
| Anxiety |  |  |  |  |  |  |  |  |  |
| STAI state | 201^a^ | 1.0(0.9 – 1.1)^a^ | 0.739^a^ | 218 | 1.03(1.00 – 1.07) | **0.047** | 202 | 1.03(0.99 – 1.06) | 0.124 |
| Period of maximum STAI state | NA | NA | NA | 218 | 0.99(0.76 – 1.30) | 0.961 | 202 | 1.0(0.8 – 1.3) | 0.786 |
| STAI trait | 201^a^ | 1.0(0.9 – 1.1)^a^ | 0.771^a^ | 218 | 1.03(0.99 – 1.07) | 0.177 | 202 | 1.03(0.99 – 1.06) | 0.158 |
| STAI trait >=40 | 201^a^ | 1.8(0.4 – 8.9) ^a^ | 0.475^a^ | 218 | 1.3(0.6 – 2.9) | 0.527 | 202 | 1.4(0.6 – 2.9) | 0.417 |
| Period of maximum STAI trait | NA | NA | NA | 218 | 1.0(0.8 – 1.3) | 0.857 | 202 | 1.1(0.8 – 1.4) | 0.653 |
| Social support |  |  |  |  |  |  |  |  |  |
| MSPSS emotional support from partner | NA | NA | NA | 163 | 0.81(0.48 – 1.36) | 0.423 | 152 | 0.86(0.50 – 1.47) | 0.576 |
| MSPSS emotional support from family | NA | NA | NA | 163 | 0.91(0.56 – 1.50) | 0.716 | 152 | 0.90(0.57 – 1.42) | 0.646 |
| MSPSS emotional support from friend | NA | NA | NA | 163 | 1.0(0.6 – 1.7) | 0.900 | 152 | 0.95(0.60 – 1.52) | 0.839 |
| MSPSS practical support from family | NA | NA | NA | 163 | 0.75(0.45 – 1.23) | 0.250 | 152 | 0.75(0.47 – 1.21) | 0.238 |
| MSPSS practical support from friend | NA | NA | NA | 163 | 0.92(0.56 – 1.51) | 0.747 | 152 | 0.95(0.59 – 1.52) | 0.831 |
| Life event |  |  |  |  |  |  |  |  |  |
| LES positive | NA | NA | NA | 158 | 0.99(0.90 – 1.09) | 0.893 | 149 | 0.99(0.91 – 1.08) | 0.821 |
| LES negative | NA | NA | NA | 158 | 1.03(0.96 – 1.11) | 0.417 | 149 | 1.0(0.9 – 1.1) | 0.709 |

BDI-II: Beck Depression Inventory-II; EPDS: Edinburgh Postnatal Depression Scale; GHQ: General Health Questionnaire; LES: Life Experiences Survey; MSPSS: Multidimensional Scale of Perceived Social Support; PAQ: Pregnancy Anxiety Questionnaire; PES: General Health Questionnaire; PSS: Perceived Stress Scale; STAI: State-Trait Anxiety Inventory

CI: confidence interval; RR: risk ratioRR = 1.0 is the reference category.

Significant p value in bold

NA: not applicable (0 count /period is constant/ stress accessed at the same time point with the outcome)

^a^Month 3 post natal stress

**Supplementary Table 4.** Univariate poisson regression of SPT at 18 month.

|  | n | RR (95% CI) | p-value |
| --- | --- | --- | --- |
| Ethnicity  Chinese  Malay  Indian  Mix | 242 | 2.4(0.7 – 7.9)  1.0  1.4(0.2 – 13.9)  2.4(0.4 – 14.1) | 0.143  0.750  0.346 |
| Education ≥ 12 years | 242 | 1.7(0.4 – 7.1) | 0.459 |
| Maternal age | 242 | 1.0(0.9 – 1.1) | 0.858 |
| Maternal allergy | 242 | 1.4(0.6 – 3.1) | 0.387 |
| Parous | 241 | 0.71(0.37 – 1.36) | 0.300 |
| Male infant | 241 | 0.77(0.42 – 1.43) | 0.413 |
| Gestational age at birth | 240 | 0.91(0.76 – 1.08) | 0.268 |
| **Preconception** |  |  |  |
| General stress |  |  |  |
| GHQ | 135 | 1.0(0.9 – 1.1) | 0.868 |
| PSS | 133 | 1.03(0.97 – 1.10) | 0.329 |
| PSS >=14 | 133 | 1.9(0.8 – 4.7) | 0.161 |
| Depression |  |  |  |
| BDI | 122 | 1.01(0.96 – 1.06) | 0.744 |
| BDI >=14 | 122 | 1.1(0.4 – 2.7) | 0.824 |
| Period of maximum BDI | 122 | 0.89(0.67 – 1.18) | 0.433 |
| EPDS | 157 | 1.0(0.9 – 1.1) | 0.989 |
| EPDS>=13 | 157 | 0.76(0.23 – 2.51) | 0.654 |
| Period of maximum EPDS | 157 | 0.95(0.70 – 1.29) | 0.738 |
| Anxiety |  |  |  |
| STAI state | 157 | 1.00(0.96 – 1.04) | 0.951 |
| Period of maximum STAI state | 157 | 1.0(0.9 – 1.2) | 0.785 |
| STAI trait | 157 | 1.00(0.96 – 1.05) | 0.948 |
| STAI trait >=40 | 157 | 0.84(0.40 – 1.77) | 0.648 |
| Period of maximum STAI trait | 157 | 0.96(0.77 – 1.20) | 0.733 |
| Social support |  |  |  |
| MSPSS emotional support from partner | 134 | 1.0(0.7 – 1.6) | 0.891 |
| MSPSS emotional support from family | 134 | 0.92(0.65 – 1.32) | 0.665 |
| MSPSS emotional support from friend | 134 | 1.1(0.7 – 1.6) | 0.782 |
| MSPSS practical support from family | 134 | 1.0(0.7 – 1.5) | 0.883 |
| MSPSS practical support from friend | 134 | 1.0(0.7 – 1.6) | 0.847 |
| Life event |  |  |  |
| LES positive | 124 | 1.1(1.0 – 1.1) | **0.033** |
| LES negative | 124 | 0.98(0.90 – 1.06) | 0.593 |
| **Pregnancy** |  |  |  |
| General stress |  |  |  |
| PES Hassles/Uplifts frequency ratio | 217 | 0.54(0.24 – 1.23) | 0.141 |
| Period of maximum PES Hassles/Uplifts frequency ratio | 217 | 1.00(0.97 – 1.03) | 0.980 |
| PES Hassles/Uplifts intensity ratio | 217 | 0.84(0.29 – 2.39) | 0.743 |
| Period of maximum PES Hassles/Uplifts intensity ratio | 217 | 0.99(0.97 – 1.02) | 0.708 |
| PSS | 216 | 0.98(0.92 – 1.04) | 0.453 |
| PSS |  |  |  |
| 0-13  14-26  >=27 | 216 | 1.0  0.65(0.31 – 1.36)  0.82(0.18 – 3.73) | 0.250  0.796 |
| Period of maximum PSS | 216 | 1.01(0.98 – 1.04) | 0.654 |
| Depression |  |  |  |
| BDI | 228 | 1.03(0.99 – 1.07) | 0.156 |
| BDI  0-13  14-19  >=20 | 228 | 1.0  1.1(0.5 – 2.5)  1.3(0.6 – 3.1) | 0.835  0.528 |
| Period of maximum BDI | 228 | 1.01(0.98 – 1.04) | 0.613 |
| EPDS | 229 | 1.05(0.99 – 1.12) | 0.125 |
| EPDS>=13 | 229 | 1.2(0.5 – 2.6) | 0.698 |
| Period of maximum EPDS | 229 | 0.99(0.97 – 1.02) | 0.635 |
| Anxiety |  |  |  |
| STAI state | 230 | 1.02(0.99 – 1.05) | 0.254 |
| Period of maximum STAI state | 230 | 0.97(0.95 – 1.00) | 0.054 |
| STAI trait | 230 | 1.01(0.98 – 1.04) | 0.568 |
| STAI trait >=40 | 230 | 1.9(1.0 – 3.9) | 0.059 |
| Period of maximum STAI trait | 230 | 0.99(0.97 – 1.02) | 0.593 |
| PAQ | 213 | 1.0(0.6 – 1.8) | 0.915 |
| Period of maximum PAQ | 213 | 0.98(0.95 – 1.01) | 0.157 |
| Social support |  |  |  |
| MSPSS emotional support from partner | 213 | 0.96(0.54 – 1.73) | 0.900 |
| Period of maximum MSPSS emotional support from partner | 213 | 1.02(0.98 – 1.07) | 0.318 |
| MSPSS emotional support from family | 213 | 1.5(0.9 – 2.7) | 0.125 |
| Period of maximum MSPSS emotional support from family | 213 | 1.02(0.98 – 1.06) | 0.244 |
| MSPSS emotional support from friend | 213 | 1.5(0.9 – 2.4) | 0.122 |
| Period of maximum MSPSS emotional support from friend | 213 | 1.01(0.98 – 1.05) | 0.398 |
| MSPSS practical support from family | 213 | 1.4(0.8 – 2.4) | 0.196 |
| Period of maximum MSPSS practical support from family | 213 | 1.01(0.98 – 1.04) | 0.569 |
| MSPSS practical support from friend | 213 | 1.3(0.8 – 1.9) | 0.315 |
| Period of maximum MSPSS practical support from friend | 213 | 1.04(1.00 – 1.09) | **0.043** |
| **Post natal** |  |  |  |
| General stress |  |  |  |
| PSS | 129 | 0.97(0.90 – 1.04) | 0.430 |
| PSS >=14 | 129 | 1.1(0.5 – 2.8) | 0.763 |
| Depression |  |  |  |
| BDI | 184 | 1.01(0.98 – 1.05) | 0.416 |
| BDI  0-13  14-19  >=20 | 184 | 1.0  1.8(0.7 – 4.4)  0.96(0.33 – 2.78) | 0.211  0.937 |
| Period of maximum BDI | 184 | 1.0(0.8 – 1.3) | 0.712 |
| EPDS | 184 | 1.0(0.9 – 1.1) | 0.880 |
| EPDS>=13 | 184 | 0.66(0.20 – 2.16) | 0.490 |
| Period of maximum EPDS | 184 | 0.86(0.68 – 1.08) | 0.195 |
| Anxiety |  |  |  |
| STAI state | 184 | 1.00(0.97 – 1.03) | 0.840 |
| Period of maximum STAI state | 184 | 0.84(0.66 – 1.07) | 0.166 |
| STAI trait | 183 | 1.01(0.97 – 1.04) | 0.707 |
| STAI trait >=40 | 183 | 1.4(0.7 – 2.9) | 0.326 |
| Period of maximum STAI trait | 183 | 0.90(0.71 – 1.14) | 0.367 |
| Social support |  |  |  |
| MSPSS emotional support from partner | 134 | 1.2(0.7 – 2.1) | 0.586 |
| MSPSS emotional support from family | 134 | 1.3(0.8 – 2.2) | 0.275 |
| MSPSS emotional support from friend | 133 | 1.1(0.7 – 1.7) | 0.691 |
| MSPSS practical support from family | 134 | 1.1(0.7 – 1.7) | 0.717 |
| MSPSS practical support from friend | 134 | 1.1(0.7 – 1.7) | 0.671 |
| Life event |  |  |  |
| LES positive | 131 | 0.99(0.91 – 1.07) | 0.754 |
| LES negative | 131 | 0.99(0.91 – 1.07) | 0.711 |

BDI-II: Beck Depression Inventory-II; EPDS: Edinburgh Postnatal Depression Scale; GHQ: General Health Questionnaire; LES: Life Experiences Survey; MSPSS: Multidimensional Scale of Perceived Social Support; PAQ: Pregnancy Anxiety Questionnaire; PES: General Health Questionnaire; PSS: Perceived Stress Scale; STAI: State-Trait Anxiety Inventory

CI: confidence interval; RR: risk ratio

RR = 1.0 is the reference category.

Significant p value in bold.

**Supplementary Table 5.** Multivariate poisson regression of eczema by 6, 12 and 18 months.

|  | 6 months | | | 12 months | | | 18 months | | |
| --- | --- | --- | --- | --- | --- | --- | --- | --- | --- |
|  | n | RR (95% CI) | p-value^b^ | n | RR (95% CI) | p-value^b^ | n | RR (95% CI) | p-value^b^ |
| **Preconception** |  |  |  |  |  |  |  |  |  |
| General distress |  |  |  |  |  |  |  |  |  |
| GHQ | 160 | 1.0(0.9 – 1.1) | 0.499 | 150 | 1.05(0.98 – 1.13) | 0.138 | 144 | 1.03(0.97 – 1.10) | 0.334 |
| PSS | 159 | 1.04(0.96 – 1.11) | 0.355 | 149 | 1.02(0.96 – 1.09) | 0.459 | 143 | 1.02(0.96 – 1.08) | 0.514 |
| PSS >=14 | 159 | 1.3(0.6 – 2.8) | 0.552 | 149 | 0.97(0.50 – 1.88) | 0.930 | 143 | 1.0(0.5 – 1.9) | 0.952 |
| Depression |  |  |  |  |  |  |  |  |  |
| BDI | 153 | 0.98(0.93 – 1.04) | 0.506 | 141 | 1.00(0.96 – 1.04) | 0.920 | 134 | 1.00(0.97 – 1.04) | 0.839 |
| BDI  0-13  14-19  >=20 | 153 | 1.0  0.77(0.10 – 5.81)  0.47(0.11 – 2.04) | 0.798  0.316 | 141 | 1.0  0.63(0.14 – 2.90)  0.96(0.39 – 2.37) | 0.553  0.926 | 134 | 1.0  0.61(0.14 – 2.67)  1.1(0.5 – 2.4) | 0.513  0.905 |
| EPDS | 190 | 0.98(0.90 – 1.07) | 0.666 | 176 | 1.02(0.95 – 1.10) | 0.516 | 169 | 1.03(0.96 – 1.10) | 0.470 |
| EPDS>=13 | 190 | 1.3(0.5 – 3.5) | 0.577 | 176 | 1.4(0.7 – 3.1) | 0.367 | 169 | 1.7(0.8 – 3.4) | 0.156 |
| Anxiety |  |  |  |  |  |  |  |  |  |
| STAI state | 189 | 1.00(0.96 – 1.04) | 0.909 | 175 | 1.00(0.97 – 1.03) | 0.913 | 168 | 1.00(0.97 – 1.03) | 0.977 |
| STAI trait | 190 | 0.99(0.95 – 1.04) | 0.787 | 176 | 1.00(0.97 – 1.04) | 0.792 | 169 | 1.00(0.97 – 1.03) | 0.962 |
| STAI trait >=40 | 190 | 0.86(0.42 – 1.75) | 0.680 | 176 | 0.84(0.47 – 1.52) | 0.569 | 169 | 0.82(0.47 – 1.44) | 0.490 |
| Social support |  |  |  |  |  |  |  |  |  |
| MSPSS emotional support from partner | 159 | 1.5(0.8 – 2.8) | 0.184 | 149 | 1.1(0.7 – 1.6) | 0.709 | 143 | 1.1(0.8 – 1.7) | 0.523 |
| MSPSS emotional support from family | 159 | 1.0(0.7 – 1.6) | 0.822 | 149 | 0.85(0.63 – 1.14) | 0.279 | 143 | 0.90(0.67 – 1.20) | 0.476 |
| MSPSS emotional support from friend | 159 | 0.96(0.63 – 1.47) | 0.855 | 149 | 0.97(0.68 – 1.38) | 0.877 | 143 | 0.99(0.70 – 1.40) | 0.955 |
| MSPSS practical support from family | 159 | 1.1(0.8 – 1.7) | 0.549 | 149 | 0.94(0.69 – 1.27) | 0.676 | 143 | 0.98(0.73 – 1.32) | 0.907 |
| MSPSS practical support from friend | 159 | 1.1(0.7 – 1.6) | 0.788 | 149 | 1.1(0.8 – 1.5) | 0.663 | 143 | 1.1(0.8 – 1.5) | 0.659 |
| Life event |  |  |  |  |  |  |  |  |  |
| LES positive | 151 | 1.0(0.9 – 1.1) | 0.971 | 140 | 0.98(0.92 – 1.03) | 0.418 | 134 | 0.99(0.94 – 1.04) | 0.706 |
| LES negative | 151 | 0.91(0.81 – 1.02) | 0.122 | 140 | 0.97(0.90 – 1.04) | 0.381 | 134 | 0.97(0.91 – 1.04) | 0.467 |
| **Pregnancy** |  |  |  |  |  |  |  |  |  |
| General distress |  |  |  |  |  |  |  |  |  |
| PES Hassles/Uplifts frequency ratio | 248 | 0.57(0.27 – 1.19) | 0.135 | 237 | 0.74(0.44 – 1.23) | 0.241 | 230 | 0.77(0.49 – 1.21) | 0.253 |
| PES Hassles/Uplifts intensity ratio | 248 | 0.77(0.27 – 2.21) | 0.628 | 237 | 0.57(0.23 – 1.42) | 0.230 | 230 | 0.60(0.26 – 1.35) | 0.218 |
| PSS | 247 | 1.03(0.97 – 1.08) | 0.363 | 236 | 1.01(0.97 – 1.06) | 0.565 | 229 | 1.00(0.96 – 1.04) | 0.981 |
| PSS  0-13  14-26  >=27 | 247 | 1.0  1.1(0.5 – 2.3)  1.8(0.5 – 7.0) | 0.898  0.399 | 236 | 1.0  1.1(0.6 – 2.2)  1.2(0.3 – 4.4) | 0.716  0.811 | 229 | 1.0  1.0(0.6 – 1.8)  0.96(0.27 – 3.43) | 0.937  0.944 |
| Depression |  |  |  |  |  |  |  |  |  |
| BDI | 264 | 1.01(0.96 – 1.05) | 0.774 | 251 | 1.00(0.96 – 1.03) | 0.842 | 244 | 1.00(0.96 – 1.03) | 0.830 |
| BDI  0-13  14-19  >=20 | 264 | 1.0  1.5(0.7 – 3.3)  0.74(0.26 – 2.12) | 0.356  0.569 | 251 | 1.0  1.0(0.5 – 2.1)  0.89(0.41 – 1.9) | 0.976  0.758 | 244 | 1.0  1.2(0.7 – 2.3)  0.90(0.44 – 1.83) | 0.501  0.763 |
| EPDS | 264 | 1.0(0.9 – 1.1) | 0.658 | 251 | 1.0(0.9 – 1.1) | 0.980 | 244 | 1.0(0.9 – 1.1) | 0.926 |
| EPDS>=13 | 264 | 1.0(0.4 – 2.2) | 0.910 | 251 | 0.76(0.37 – 1.56) | 0.457 | 244 | 0.64(0.32 – 1.29) | 0.213 |
| Anxiety |  |  |  |  |  |  |  |  |  |
| STAI state | 265 | 1.02(0.99 – 1.05) | 0.232 | 252 | 1.00(0.98 – 1.03) | 0.902 | 245 | 0.99(0.97 – 1.02) | 0.643 |
| STAI trait | 265 | 1.00(0.97 – 1.04) | 0.794 | 252 | 0.99(0.97 – 1.02) | 0.664 | 245 | 0.99(0.96 – 1.02) | 0.467 |
| STAI trait >=40 | 265 | 1.3(0.7 – 2.4) | 0.402 | 252 | 1.1(0.7 – 1.9) | 0.607 | 245 | 1.1(0.7 – 1.8) | 0.568 |
| PAQ | 244 | 0.76(0.44 – 1.32) | 0.335 | 233 | 0.68(0.43 – 1.08) | 0.100 | 226 | 0.66(0.43 – 1.01) | 0.053 |
| Social support |  |  |  |  |  |  |  |  |  |
| MSPSS emotional support from partner | 245 | 0.99(0.56 – 1.76) | 0.982 | 234 | 0.89(0.56 – 1.42) | 0.632 | 227 | 1.0(0.6 – 1.6) | 0.907 |
| MSPSS emotional support from family | 245 | 1.0(0.7 – 1.6) | 0.902 | 234 | 0.88(0.64 – 1.21) | 0.438 | 227 | 1.0(0.8 – 1.4) | 0.819 |
| MSPSS emotional support from friend | 245 | 0.97(0.65 – 1.44) | 0.870 | 234 | 1.0(0.7 – 1.4) | 0.921 | 227 | 1.0(0.8 – 1.4) | 0.953 |
| MSPSS practical support from family | 245 | 1.1(0.7 – 1.8) | 0.622 | 234 | 0.91(0.65 – 1.27) | 0.582 | 227 | 1.0(0.8 – 1.4) | 0.856 |
| MSPSS practical support from friend | 245 | 1.0(0.7 – 1.5) | 0.871 | 234 | 1.04(0.76 – 1.43) | 0.794 | 227 | 1.1(0.8 – 1.5) | 0.539 |
| **Postnatal^c^** |  |  |  |  |  |  |  |  |  |
| General distress |  |  |  |  |  |  |  |  |  |
| PSS | NA | NA | NA | 156 | 0.98(0.93 – 1.04) | 0.545 | 151 | 0.99(0.94 – 1.04) | 0.594 |
| PSS  0-13  14-26  >=27 | NA | NA | NA | 156 | 1.0  1.1(0.6 – 2.2)  0.56(0.07 – 4.76) | 0.767  0.595 | 151 | 1.0  0.89(0.50 – 1.61)  0.63(0.08 – 5.07) | 0.712  0.663 |
| Depression |  |  |  |  |  |  |  |  |  |
| BDI | 200^a^ | 0.99(0.94 – 1.04)^a^ | 0.688^a^ | 224 | 1.00(0.96 – 1.03) | 0.823 | 218 | 1.00(0.97 – 1.03) | 0.994 |
| BDI  0-13  14-19  >=20 | 200^a^ | 1.0  0.71(0.16 – 3.08)^a^  0.52(0.12 – 2.20)^a^ | 0.647^a^  0.372^a^ | 224 | 1.0  0.80(0.31 – 2.04)  0.95(0.42 – 2.13) | 0.634  0.901 | 218 | 1.0  1.1(0.5 – 2.4)  0.92(0.41 – 2.06) | 0.767  0.845 |
| EPDS | 200^a^ | ­ | 0.216^a^ | 224 | 1.03(0.98 – 1.09) | 0.247 | 218 | 1.02(0.97 – 1.08) | 0.356 |
| EPDS>=13 | 200^a^ | 0.62(0.15 – 2.65)^a^ | 0.522^a^ | 224 | 1.1(0.5 – 2.4) | 0.822 | 218 | 1.1(0.5 – 2.2) | 0.813 |
| Anxiety |  |  |  |  |  |  |  |  |  |
| STAI state | 200^a^ | 1.02(0.99 – 1.05)^a^ | 0.253^a^ | 223 | 1.01(0.98 – 1.03) | 0.510 | 217 | 1.01(0.98 – 1.03) | 0.604 |
| STAI trait | 200^a^ | 1.01(0.98 – 1.05)^a^ | 0.441^a^ | 223 | 1.01(0.98 – 1.03) | 0.707 | 217 | 1.00(0.98 – 1.03) | 0.869 |
| STAI trait >=40 | 200^a^ | 1.5(0.7 – 3.0)^a^ | 0.291^a^ | 223 | 1.2(0.7 – 2.1) | 0.429 | 217 | 1.1(0.7 – 1.8) | 0.656 |
| Social support |  |  |  |  |  |  |  |  |  |
| MSPSS emotional support from partner | NA | NA | NA | 165 | 0.80(0.57 – 1.12) | 0.188 | 160 | 0.87(0.63 – 1.21) | 0.407 |
| MSPSS emotional support from family | NA | NA | NA | 165 | 0.92(0.66 – 1.27) | 0.606 | 160 | 1.0(0.7 – 1.4) | 0.987 |
| MSPSS emotional support from friend | NA | NA | NA | 164 | 1.1(0.8 – 1.5) | 0.615 | 159 | 1.1(0.8 – 1.5) | 0.613 |
| MSPSS practical support from family | NA | NA | NA | 165 | 0.91(0.64 – 1.3) | 0.607 | 160 | 0.94(0.68 – 1.30) | 0.725 |
| MSPSS practical support from friend | NA | NA | NA | 165 | 1.0(0.8 – 1.4) | 0.791 | 160 | 0.99(0.74 – 1.32) | 0.924 |
| Life event |  |  |  |  |  |  |  |  |  |
| LES positive | NA | NA | NA | 159 | 1.01(0.95 – 1.08) | 0.702 | 156 | 1.02(0.97 – 1.08) | 0.453 |
| LES negative | NA | NA | NA | 159 | 0.98(0.92 – 1.04) | 0.460 | 156 | 0.98(0.93 – 1.04) | 0.474 |

BDI-II: Beck Depression Inventory-II; EPDS: Edinburgh Postnatal Depression Scale; GHQ: General Health Questionnaire; LES: Life Experiences Survey; MSPSS: Multidimensional Scale of Perceived Social Support; PAQ: Pregnancy Anxiety Questionnaire; PES: General Health Questionnaire; PSS: Perceived Stress Scale; STAI: State-Trait Anxiety Inventory

CI: confidence interval, RR: risk ratio

RR = 1.0 is the reference category.

NA: not applicable (stress accessed at the same time point with the outcome)

^a^Month 3 postnatal stress

^b^adjusted for period of maximum stress (if stress accessed at several time points), ethnicity, maternal age at birth, length of education, parity, smoking during pregnancy, maternal history of allergy, infant sex and gestational age at birth.

^c^adjusted for period of maximum stress (if stress accessed at several time points), ethnicity, maternal age at birth, length of education, parity, maternal history of allergy, infant sex and gestational age at birth.

Benjamini-Hochberg correction with false discovery rate at 0.45 and n=155 was applied.

**Supplementary Table 6.** Multivariate poisson regression of rhinitis by 6, 12 and 18 months.

|  | 6 months | | | 12 months | | | 18 months | | |
| --- | --- | --- | --- | --- | --- | --- | --- | --- | --- |
|  | n | RR (95% CI) | p-value^b^ | n | RR (95% CI) | p-value^b^ | n | RR (95% CI) | p-value^b^ |
| **Preconception** |  |  |  |  |  |  |  |  |  |
| General distress |  |  |  |  |  |  |  |  |  |
| GHQ | 162 | 1.03(0.98 – 1.09) | 0.258 | 155 | 1.04(0.99 – 1.09) | 0.104 | 148 | 1.04(0.99 – 1.08) | 0.104 |
| PSS | 161 | 1.03(0.98 – 1.08) | 0.290 | 154 | 1.04(0.99 – 1.08) | 0.116 | 147 | 1.03(0.99 – 1.07) | 0.148 |
| PSS  0-13  14-26  >=27 | 161 | 1.0  1.0(0.6 – 1.8)  0.85(0.1 – 6.89) | 0.952  0.878 | 154 | 1.0  1.3(0.7 – 2.1)  0.73(0.09 – 5.77) | 0.392  0.761 | 147 | 1.0  1.2(0.7 – 2.0)  1.3(0.3 – 5.9) | 0.475  0.738 |
| Depression |  |  |  |  |  |  |  |  |  |
| BDI | 156 | 1.02(0.98 – 1.05) | 0.362 | 145 | 1.02(0.99 – 1.05) | 0.189 | 136 | 1.03(1.00 – 1.05) | 0.074 |
| BDI  0-13  14-19  >=20 | 156 | 1.0  1.4(0.5 – 3.8)  1.4(0.7 – 3.1) | 0.550  0.350 | 145 | 1.0  1.3(0.5 – 3.6)  1.5(0.8 – 2.8) | 0.609  0.234 | 136 | 1.0  1.8(0.8 – 4.2)  1.5(0.8 – 2.8) | 0.185  0.158 |
| EPDS | 192 | 1.05(0.99 – 1.11) | 0.144 | 181 | 1.04(0.99 – 1.10) | 0.112 | 172 | 1.03(0.98 – 1.09) | 0.188 |
| EPDS>=13 | 192 | 1.5(0.8 – 3.0) | 0.245 | 181 | 1.7(0.9 – 3.0) | 0.089 | 172 | 1.5(0.9 – 2.6) | 0.127 |
| Anxiety |  |  |  |  |  |  |  |  |  |
| STAI state | 191 | 1.01(0.98 – 1.04) | 0.496 | 180 | 1.01(0.98 – 1.03) | 0.455 | 171 | 1.01(0.98 – 1.03) | 0.564 |
| STAI trait | 192 | 1.02(0.99 – 1.05) | 0.323 | 181 | 1.02(0.99 – 1.05) | 0.138 | 172 | 1.02(0.99 – 1.04) | 0.175 |
| STAI trait >=40 | 192 | 1.6(0.9 – 2.6) | 0.082 | 181 | 1.6(1.0 – 2.5) | 0.046 | 172 | 1.3(0.9 – 2.0) | 0.179 |
| Social support |  |  |  |  |  |  |  |  |  |
| MSPSS emotional support from partner | 161 | 1.0(0.7 – 1.4) | 0.854 | 154 | 0.96(0.73 – 1.27) | 0.777 | 147 | 0.99(0.76 – 1.28) | 0.927 |
| MSPSS emotional support from family | 161 | 0.92(0.71 – 1.19) | 0.526 | 154 | 0.91(0.72 – 1.15) | 0.444 | 147 | 0.93(0.74 – 1.16) | 0.506 |
| MSPSS emotional support from friend | 161 | 0.99(0.73 – 1.33) | 0.922 | 154 | 0.89(0.69 – 1.16) | 0.390 | 147 | 0.97(0.76 – 1.24) | 0.808 |
| MSPSS practical support from family | 161 | 0.97(0.75 – 1.25) | 0.796 | 154 | 1.0(0.8 – 1.3) | 0.998 | 147 | 1.0(0.8 – 1.2) | 0.962 |
| MSPSS practical support from friend | 161 | 1.0(0.7 – 1.3) | 0.970 | 154 | 0.87(0.68 – 1.12) | 0.279 | 147 | 0.93(0.74 – 1.18) | 0.558 |
| Life event |  |  |  |  |  |  |  |  |  |
| LES positive | 153 | 1.03(0.98 – 1.08) | 0.204 | 146 | 1.00(0.96 – 1.05) | 0.861 | 139 | 1.00(0.96 – 1.04) | 0.912 |
| LES negative | 153 | 1.01(0.95 – 1.07) | 0.773 | 146 | 1.02(0.97 – 1.07) | 0.435 | 139 | 1.02(0.97 – 1.07) | 0.402 |
| **Pregnancy** |  |  |  |  |  |  |  |  |  |
| General distress |  |  |  |  |  |  |  |  |  |
| PES Hassles/Uplifts frequency ratio | 257 | 0.79(0.53 – 1.18) | 0.250 | 246 | 0.85(0.61 – 1.19) | 0.351 | 239 | 0.88(0.65 – 1.20) | 0.413 |
| PES Hassles/Uplifts intensity ratio | 257 | 1.2(0.6 – 2.2) | 0.654 | 246 | 1.1(0.6 – 2.0) | 0.701 | 239 | 0.96(0.55 – 1.67) | 0.875 |
| PSS | 256 | 1.01(0.97 – 1.05) | 0.548 | 245 | 1.03(1.00 – 1.07) | 0.084 | 238 | 1.02(0.99 – 1.05) | 0.232 |
| PSS  0-13  14-26  >=27 | 256 | 1.0  0.95(0.57 – 1.58)  1.3(0.5 – 3.7) | 0.834  0.586 | 245 | 1.0  1.1(0.7 – 1.7)  1.8(0.7 – 4.4) | 0.820  0.191 | 238 | 1.0  1.1(0.7 – 1.7)  1.5(0.6 – 3.6) | 0.704  0.372 |
| Depression |  |  |  |  |  |  |  |  |  |
| BDI | 273 | 1.01(0.99 – 1.04) | 0.310 | 259 | 1.02(1.00 – 1.04) | 0.062 | 251 | 1.02(0.99 – 1.04) | 0.145 |
| BDI  0-13  14-19  >=20 | 273 | 1.0  1.2(0.7 – 2.2)  1.2(0.7 – 2.2) | 0.434  0.450 | 259 | 1.0  1.4(0.9 – 2.2)  1.4(0.9 – 2.3) | 0.180  0.139 | 251 | 1.0  1.3(0.8 – 2.0)  1.3(0.8 – 2.0) | 0.282  0.271 |
| EPDS | 273 | 1.03(0.99 – 1.08) | 0.116 | 259 | 1.05(1.01 – 1.08) | 0.018 | 251 | 1.03(0.99 – 1.07) | 0.120 |
| EPDS>=13 | 273 | 1.3(0.8 – 2.2) | 0.282 | 259 | 1.5(1.0 – 2.2) | 0.064 | 251 | 1.3(0.9 – 1.9) | 0.224 |
| Anxiety |  |  |  |  |  |  |  |  |  |
| STAI state | 274 | 1.01(0.99 – 1.03) | 0.546 | 260 | 1.01(0.99 – 1.03) | 0.212 | 252 | 1.00(0.99 – 1.02) | 0.634 |
| STAI trait | 274 | 1.01(0.99 – 1.03) | 0.376 | 260 | 1.02(1.00 – 1.04) | 0.110 | 252 | 1.01(0.99 – 1.03) | 0.294 |
| STAI trait >=40 | 274 | 1.3(0.8 – 1.9) | 0.259 | 260 | 1.3(0.9 – 1.9) | 0.148 | 252 | 1.1(0.8 – 1.6) | 0.436 |
| PAQ | 253 | 1.5(1.0 – 2.1) | 0.026 | 242 | 1.4(1.1 – 1.9) | 0.023 | 235 | 1.3(1.0 – 1.8) | 0.043 |
| Social support |  |  |  |  |  |  |  |  |  |
| MSPSS emotional support from partner | 254 | 1.0(0.7 – 1.5) | 0.877 | 243 | 0.96(0.70 – 1.33) | 0.818 | 236 | 1.0(0.7 – 1.4) | 0.990 |
| MSPSS emotional support from family | 254 | 0.80(0.61 – 1.05) | 0.112 | 243 | 0.82(0.65 – 1.04) | 0.095 | 236 | 0.88(0.70 – 1.10) | 0.251 |
| MSPSS emotional support from friend | 254 | 0.91(0.71 – 1.17) | 0.473 | 243 | 0.90(0.72 – 1.13) | 0.363 | 236 | 0.95(0.77 – 1.17) | 0.636 |
| MSPSS practical support from family | 254 | 0.87(0.66 – 1.14) | 0.303 | 243 | 0.84(0.67 – 1.07) | 0.154 | 236 | 0.84(0.67 – 1.05) | 0.128 |
| MSPSS practical support from friend | 254 | 0.86(0.67 – 1.09) | 0.212 | 243 | 0.90(0.72 – 1.11) | 0.322 | 236 | 0.95(0.78 – 1.17) | 0.636 |
| **Postnatal^c^** |  |  |  |  |  |  |  |  |  |
| General distress |  |  |  |  |  |  |  |  |  |
| PSS | NA | NA | NA | 163 | 1.01(0.96 – 1.05) | 0.744 | 158 | 1.00(0.96 – 1.05) | 0.850 |
| PSS  0-13  14-26  >=27 | NA | NA | NA | 163 | 1.0  0.91(0.53 – 1.56)  1.5(0.4 – 5.5) | 0.732  0.529 | 158 | 1.0  0.90(0.53 – 1.50)  1.5(0.5 – 4.4) | 0.676  0.504 |
| Depression |  |  |  |  |  |  |  |  |  |
| BDI | 206^a^ | 1.02(0.99 – 1.04) ^a^ | 0.216^a^ | 233 | 1.01(0.99 – 1.04) | 0.286 | 225 | 1.01(0.99 – 1.03) | 0.221 |
| BDI  0-13  14-19  >=20 | 206^a^ | 1.0  1.8(0.9 – 3.7)^a^  1.3(0.6 – 2.7)^a^ | 0.108^a^  0.500^a^ | 233 | 1.0  1.3(0.8 – 2.3)  1.1(0.6 – 1.9) | 0.294  0.707 | 225 | 1.0  1.3(0.7 – 2.2)  1.2(0.7 – 2.0) | 0.387  0.514 |
| EPDS | 206^a^ | 1.01(0.96 – 1.06)^a^ | 0.785^a^ | 233 | 0.99(0.95 – 1.03) | 0.672 | 225 | 1.00(0.96 – 1.03) | 0.812 |
| EPDS>=13 | 206^a^ | 0.93(0.39 – 2.19)^a^ | 0.860^a^ | 233 | 0.81(0.44 – 1.48) | 0.495 | 225 | 0.91(0.53 – 1.56) | 0.725 |
| Anxiety | 206^a^ |  |  |  |  |  |  |  |  |
| STAI state | 206^a^ | 1.01(0.99 – 1.03)^a^ | 0.258^a^ | 232 | 1.00(0.99 – 1.02) | 0.603 | 224 | 1.00(0.99 – 1.02) | 0.827 |
| STAI trait | 206^a^ | 1.01(0.99 – 1.04)^a^ | 0.259^a^ | 232 | 1.01(0.99 – 1.02) | 0.578 | 224 | 1.00(0.98 – 1.02) | 0.753 |
| STAI trait >=40 | 206^a^ | 1.7(1.0 – 2.8)^a^ | 0.041^a^ | 232 | 1.3(0.9 – 1.9) | 0.218 | 224 | 1.2(0.8 – 1.7) | 0.436 |
| Social support |  |  |  |  |  |  |  |  |  |
| MSPSS emotional support from partner | NA | NA | NA | 170 | 0.85(0.64 – 1.12) | 0.246 | 165 | 0.90(0.69 – 1.17) | 0.421 |
| MSPSS emotional support from family | NA | NA | NA | 170 | 0.91(0.71 – 1.16) | 0.435 | 165 | 0.92(0.72 – 1.16) | 0.481 |
| MSPSS emotional support from friend | NA | NA | NA | 169 | 0.90(0.71 – 1.16) | 0.418 | 164 | 0.94(0.74 – 1.19) | 0.617 |
| MSPSS practical support from family | NA | NA | NA | 170 | 0.96(0.73 – 1.25) | 0.740 | 165 | 0.98(0.76 – 1.27) | 0.889 |
| MSPSS practical support from friend | NA | NA | NA | 170 | 0.85(0.66 – 1.09) | 0.194 | 165 | 0.91(0.71 – 1.15) | 0.426 |
| Life event |  |  |  |  |  |  |  |  |  |
| LES positive | NA | NA | NA | 164 | 1.01(0.97 – 1.06) | 0.505 | 160 | 1.01(0.97 – 1.05) | 0.734 |
| LES negative | NA | NA | NA | 164 | 1.02(0.98 – 1.06) | 0.309 | 160 | 1.01(0.98 – 1.05) | 0.479 |

BDI-II: Beck Depression Inventory-II; EPDS: Edinburgh Postnatal Depression Scale; GHQ: General Health Questionnaire; LES: Life Experiences Survey; MSPSS: Multidimensional Scale of Perceived Social Support; PAQ: Pregnancy Anxiety Questionnaire; PES: General Health Questionnaire; PSS: Perceived Stress Scale; STAI: State-Trait Anxiety Inventory

CI: confidence interval, RR: risk ratio

RR = 1.0 is the reference category.

NA: not applicable (stress accessed at the same time point with the outcome)

^a^Month 3 postnatal stress

^b^adjusted for period of maximum stress (if stress accessed at several time points), ethnicity, maternal age at birth, length of education, parity, smoking during pregnancy, maternal history of allergy, infant sex and gestational age at birth.

^c^adjusted for period of maximum stress (if stress accessed at several time points), ethnicity, maternal age at birth, length of education, parity, maternal history of allergy, infant sex and gestational age at birth.

Benjamini-Hochberg correction with false discovery rate at 0.45 and n=158 was applied.

**Supplementary Table 7.** Multivariate poisson regression of SPT at 18 months.

|  | n | RR (95% CI) | p-value^a^ |
| --- | --- | --- | --- |
| **Preconception** |  |  |  |
| General distress |  |  |  |
| GHQ | 127 | 1.0(0.9 – 1.1) | 0.795 |
| PSS | 126 | 1.1(1.0 – 1.2) | 0.219 |
| PSS >=14 | 126 | 2.3(0.8 – 7.0) | 0.144 |
| Depression |  |  |  |
| BDI | 115 | 1.03(0.97 – 1.09) | 0.303 |
| BDI >=14 | 115 | 1.0(0.3 – 3.3) | 0.967 |
| EPDS | 149 | 1.1(0.9 – 1.2) | 0.354 |
| EPDS>=13 | 149 | 0.96(0.28 – 3.33) | 0.950 |
| Anxiety |  |  |  |
| STAI state | 149 | 1.01(0.97 – 1.06) | 0.644 |
| STAI trait | 149 | 1.02(0.97 – 1.07) | 0.365 |
| STAI trait >=40 | 149 | 1.3(0.6 – 3.1) | 0.501 |
| Social support |  |  |  |
| MSPSS emotional support from partner | 126 | 0.99(0.60 – 1.62) | 0.960 |
| MSPSS emotional support from family | 126 | 0.97(0.63 – 1.50) | 0.898 |
| MSPSS emotional support from friend | 126 | 1.1(0.6 – 1.8) | 0.844 |
| MSPSS practical support from family | 126 | 1.1(0.7 – 1.7) | 0.743 |
| MSPSS practical support from friend | 126 | 0.91(0.55 – 1.50) | 0.711 |
| Life event |  |  |  |
| LES positive | 117 | 1.1(1.0 – 1.2) | 0.013 |
| LES negative | 117 | 1.0(0.9 – 1.1) | 0.543 |
| **Pregnancy** |  |  |  |
| General distress |  |  |  |
| PES Hassles/Uplifts frequency ratio | 206 | 0.53(0.22 – 1.28) | 0.157 |
| PES Hassles/Uplifts intensity ratio | 206 | 0.86(0.30 – 2.50) | 0.783 |
| PSS | 205 | 0.97(0.91 – 1.03) | 0.345 |
| PSS  0-13  14-26  >=27 | 205 | 1.0  0.64(0.29 – 1.43)  0.41(0.05 – 3.52) | 0.277  0.419 |
| Depression |  |  |  |
| BDI | 217 | 1.04(0.99 – 1.08) | 0.116 |
| BDI  0-13  14-19  >=20 | 217 | 1.0  1.6(0.6 – 3.9)  1.2(0.4 – 3.2) | 0.338  0.762 |
| EPDS | 217 | 1.04(0.97 – 1.11) | 0.284 |
| EPDS>=13 | 217 | 1.1(0.4 – 2.6) | 0.891 |
| Anxiety |  |  |  |
| STAI state | 218 | 1.01(0.98 – 1.04) | 0.548 |
| STAI trait | 218 | 1.01(0.97 – 1.04) | 0.748 |
| STAI trait >=40 | 218 | 1.7(0.8 – 3.5) | 0.155 |
| PAQ | 202 | 0.92(0.50 – 1.71) | 0.800 |
| Social support |  |  |  |
| MSPSS emotional support from partner | 202 | 0.88(0.44 – 1.78) | 0.726 |
| MSPSS emotional support from family | 202 | 1.5(0.8 – 2.7) | 0.183 |
| MSPSS emotional support from friend | 202 | 1.5(0.9 – 2.5) | 0.154 |
| MSPSS practical support from family | 202 | 1.5(0.8 – 2.7) | 0.176 |
| MSPSS practical support from friend | 202 | 1.2(0.7 – 2.0) | 0.459 |
| **Postnatal^b^** |  |  |  |
| General distress |  |  |  |
| PSS | 129 | 0.97(0.90 – 1.05) | 0.449 |
| PSS >=14 | 129 | 1.1(0.4 – 3.0) | 0.782 |
| Depression |  |  |  |
| BDI | 183 | 1.02(0.98 – 1.06) | 0.316 |
| BDI  0-13  14-19  >=20 | 183 | 1.0  1.8(0.7 – 4.6)  1.1(0.4 – 3.1) | 0.217  0.924 |
| EPDS | 183 | 1.0(0.9 – 1.1) | 0.979 |
| EPDS>=13 | 183 | 0.73(0.21 – 2.55) | 0.624 |
| Anxiety |  |  |  |
| STAI state | 183 | 1.00(0.97 – 1.04) | 0.881 |
| STAI trait | 182 | 1.01(0.97 – 1.04) | 0.709 |
| STAI trait >=40 | 182 | 1.3(0.6 – 2.7) | 0.482 |
| Social support |  |  |  |
| MSPSS emotional support from partner | 134 | 1.3(0.7 – 2.4) | 0.463 |
| MSPSS emotional support from family | 134 | 1.4(0.8 – 2.3) | 0.233 |
| MSPSS emotional support from friend | 133 | 1.2(0.7 – 1.8) | 0.556 |
| MSPSS practical support from family | 134 | 1.2(0.7 – 1.9) | 0.558 |
| MSPSS practical support from friend | 134 | 1.2(0.7 – 1.8) | 0.539 |
| Life event |  |  |  |
| LES positive | 131 | 0.98(0.90 – 1.08) | 0.729 |
| LES negative | 131 | 0.99(0.91 – 1.08) | 0.811 |

BDI-II: Beck Depression Inventory-II; EPDS: Edinburgh Postnatal Depression Scale; GHQ: General Health Questionnaire; LES: Life Experiences Survey; MSPSS: Multidimensional Scale of Perceived Social Support; PAQ: Pregnancy Anxiety Questionnaire; PES: General Health Questionnaire; PSS: Perceived Stress Scale; STAI: State-Trait Anxiety Inventory

CI: confidence interval, RR: risk ratio, SPT: skin prick test

RR = 1.0 is the reference category.

^a^adjusted for period of maximum distress (if distress accessed at several time points), ethnicity, maternal age at birth, length of education, parity, smoking during pregnancy, maternal history of allergy, infant sex and gestational age at birth.

^b^adjusted for period of maximum distress (if distress accessed at several time points), ethnicity, maternal age at birth, length of education, parity, maternal history of allergy, infant sex and gestational age at birth.

Benjamini-Hochberg correction with false discovery rate at 0.45 and n= 53 was applied.
